# Mitochondrial pyruvate transport regulates presynaptic metabolism and neurotransmission

**DOI:** 10.1126/sciadv.adp7423

**Published:** 2024-11-15

**Authors:** Anupama Tiwari, Jongyun Myeong, Arsalan Hashemiaghdam, Marion I. Stunault, Hao Zhang, Xiangfeng Niu, Marissa A. Laramie, Jasmin Sponagel, Leah P. Shriver, Gary J. Patti, Vitaly A. Klyachko, Ghazaleh Ashrafi

**Affiliations:** ^1^ Department of Cell Biology and Physiology, Washington University School of Medicine, St. Louis, MO, USA.; ^2^ Department of Chemistry, Department of Medicine, Center for Mass Spectrometry and Metabolic Tracing, Washington University in St. Louis, St. Louis, MO, USA.; ^3^ Needleman Center for Neurometabolism and Axonal Therapeutics, Washington University School of Medicine, St. Louis, MO, USA.

## Abstract

Glucose has long been considered the primary fuel source for the brain. However, glucose levels fluctuate in the brain during sleep or circuit activity, posing major metabolic stress. Here, we demonstrate that the mammalian brain uses pyruvate as a fuel source, and pyruvate can support neuronal viability in the absence of glucose. Nerve terminals are sites of metabolic vulnerability, and we show that mitochondrial pyruvate uptake is a critical step in oxidative ATP production in hippocampal terminals. We find that the mitochondrial pyruvate carrier is post-translationally modified by lysine acetylation, which, in turn, modulates mitochondrial pyruvate uptake. Our data reveal that the mitochondrial pyruvate carrier regulates distinct steps in neurotransmission, namely, the spatiotemporal pattern of synaptic vesicle release and the efficiency of vesicle retrieval—functions that have profound implications for synaptic plasticity. In summary, we identify pyruvate as a potent neuronal fuel and mitochondrial pyruvate uptake as a critical node for the metabolic control of neurotransmission in hippocampal terminals.

## Teaser

The brain uses pyruvate as an energy source, and mitochondrial pyruvate metabolism in nerve terminals controls neurotransmission.

## INTRODUCTION

The brain requires a constant, ready source of energy to function properly. When metabolic processes supporting energy generation fail, for example, secondary to ischemic stroke or uncontrolled diabetes, loss of cognitive function follows rapidly (*1*, *2*). Paradoxically, even in a healthy brain, access to glucose, its canonical energy source, is relatively unreliable. Glucose concentration in the brain interstitial fluid is low (~1 mM) (*3*) and is further depleted by bouts of high neuronal activity (*4*, *5*) during fasting and sleep (*6*). Neurons consume ~40% of total adenosine 5′-triphosphate (ATP) in the cortex to sustain synaptic transmission (*7*), the signaling process underpinning cognition. The fluctuating levels of glucose in the brain, coupled with limited glycogen storage in this organ, suggest that neurons would have to rely on alternative energy sources to sustain synaptic transmission (*8*). These alternative fuels primarily include amino acids, ketone bodies, pyruvate, and its derivative lactate.

Pyruvate can be generated from the glycolytic breakdown of glucose or catabolism of amino acids such as alanine, serine, and threonine. There are two major routes for neurons to acquire pyruvate: from the blood supply or as lactate released by neighboring astrocytes. Although the physiological relevance of pyruvate delivery to the brain remains unclear, it is known that endothelial cells and astrocytic end feet forming the blood-brain barrier express monocarboxylate transporters, which can transport pyruvate from blood to the brain interstitial fluid (*9*). Magnetic resonance imaging has shown that pyruvate can enter the human brain from the circulation (*10*), supporting the notion that pyruvate could serve as an energy source for the brain (*11*–*14*). Yet, the extent to which pyruvate supports neuronal functions and the molecular mechanisms of pyruvate oxidation in neurons remain poorly understood.

Pyruvate is oxidized via the tricarboxylic acid (TCA) cycle in the mitochondrial matrix. Once taken up into neuronal cytosol by monocarboxylate transporters, pyruvate must traverse mitochondrial inner and outer membranes to gain access to TCA enzymes. The voltage-dependent anion channel makes mitochondrial outer membrane largely permeable to metabolites such as pyruvate (*15*). However, pyruvate passage through the inner mitochondrial membrane is facilitated by the mitochondrial pyruvate carrier (MPC), a multimeric complex of two subunits: MPC1 and MPC2 (*16*, *17*). By regulating pyruvate entry to the mitochondrial matrix and its access to the TCA cycle, MPC mediates a crucial branch point in energy metabolism. The physiological importance of MPC is further highlighted by its implication in several human pathologies, including cancer, cardiac hypertrophy, diabetes, and microcephaly (*18*). There is also growing interest in targeting MPC for treatment of neurological disorders such as Parkinson’s disease (*19*, *20*). However, few studies have examined MPC function in the nervous system (*21*, *22*), particularly in the metabolic control of synaptic transmission.

Mathematical models suggest that mitochondrial pyruvate uptake is a limiting step in stimulation of oxidative ATP synthesis (*23*). Yeast cells up-regulate pyruvate oxidation under aerobic growth conditions through alternative expression of MPC isoforms with enhanced transport kinetics (*24*). However, this mechanism is not conserved in mammalian cells, and it is not known whether or how MPC activity is modulated in energetically demanding cells such as neurons. In the present study, we investigated pyruvate metabolism in the brain and found its critical role in the regulation of synaptic transmission. We demonstrated that mitochondrial pyruvate transport is essential for ATP production in nerve terminals and regulates distinct steps in the synaptic vesicle (SV) cycle. We further uncovered a posttranslational mechanism for regulation of mitochondrial pyruvate transport. Together, our study establishes that mitochondrial pyruvate uptake in nerve terminals is precisely modulated to ensure the metabolic plasticity of neurotransmission.

## RESULTS

### Pyruvate is efficiently oxidized in intact brain and is a metabolic fuel for neuronal cultures

**Fig. 1.**
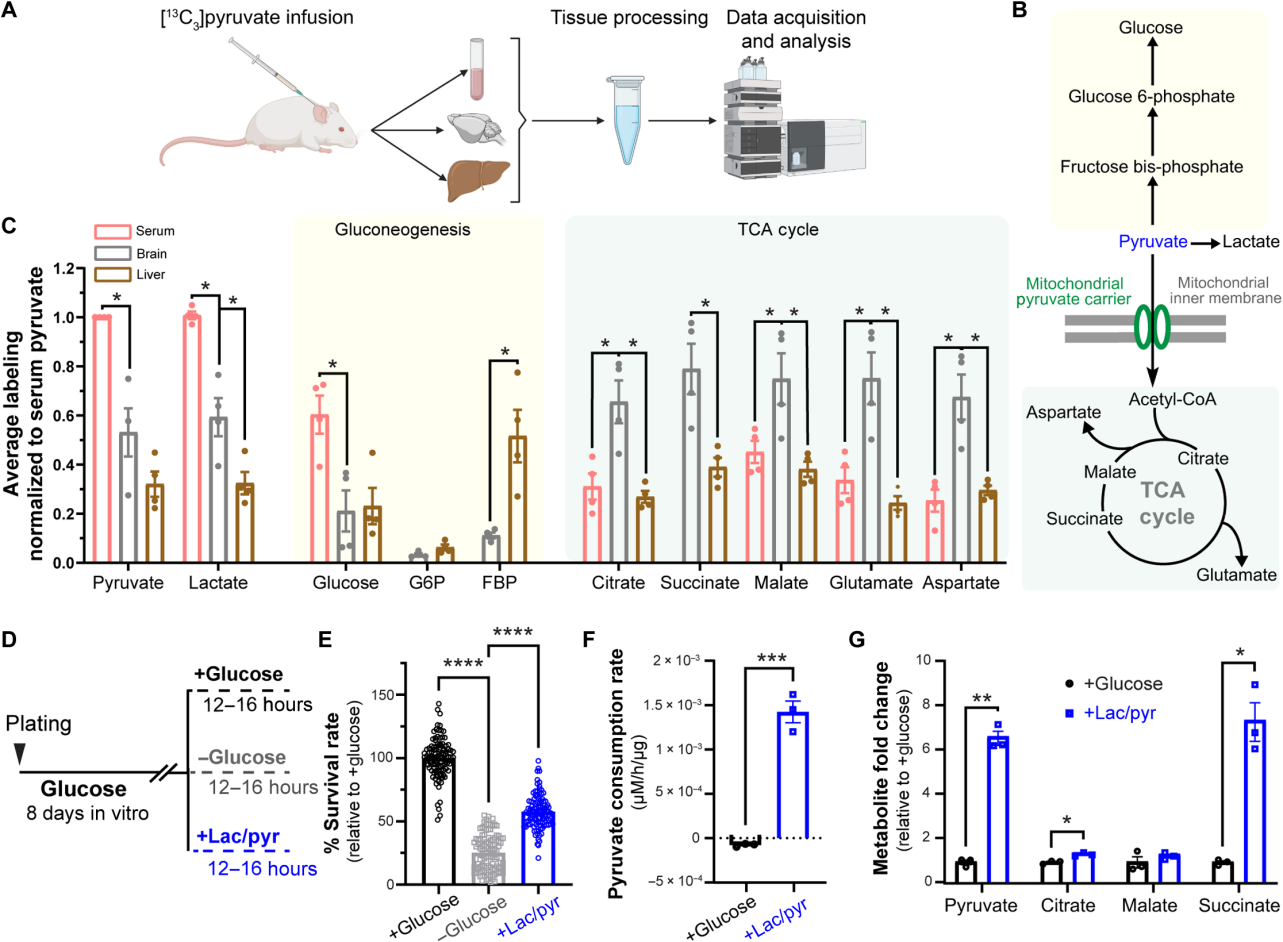
Pyruvate is efficiently oxidized in intact brain and is a metabolic fuel for primary neuronal cultures. (**A**) Schematic of ^13^C_3_ pyruvate perfusion into mouse jugular vein and its metabolic tracing in the serum, brain, and liver using LC-MS. Created with Biorender.com. (**B**) Pathways of pyruvate metabolism including gluconeogenesis and oxidation via the TCA cycle. (**C**) Enrichment of ^13^C-labeled (m + 1 to m + 6) intermediates of gluconeogenesis and the TCA cycle in different tissues. ^13^C-labeled metabolite levels were normalized to the amount of circulating tracer. Serum glucose-6-phosphate (G6P), FBP, and succinate were excluded due to lack of reliable measurement. *n* = 4 mice. (**D**) Schematic for survival analysis and metabolic profiling of primary neurons at 8 DIV supplied with various fuel types, with glucose (5 mM), no glucose, or an equimolar mix of lactate and pyruvate (5 mM each) denoted as lac/pyr. (**E**) Survival rate of neurons relative to control (+glucose). *n* = 108 (wells), 3 (cultures). (**F**) Pyruvate consumption rate (μM/hour per μg of lysate) in neurons treated as in (E). *n* = 3 (wells). (**G**) Steady-state levels of TCA metabolites in neurons treated as in (D) and plotted as fold change relative to +glucose condition = 3 (wells). Unpaired *t* test [(F) and (G)] and one-way analysis of variance (ANOVA) [(C) and (E)]. *P < 0.05, **P < 0.01, ***P < 0.001, ****P < 0.0001, *q < 0.01, **q < 0.001, and ***P < 0.0001. Error bars are SEM.

It has been previously shown that acute supplementation of cultured neurons or brain slices with pyruvate can sustain the energetics of neurotransmission (*12*–*14*). However, it is not clear to what extent chronic pyruvate supply can support neuronal survival in the absence of glucose, and whether pyruvate can serve as a bona fide fuel source for the intact brain. To examine pyruvate metabolism in the rodent brain, we performed intravenous infusion of ^13^C_3_-labeled pyruvate into the jugular vein of C57BL/6 mice that were briefly fasted (for ~5 hours) and analyzed the metabolic fate of pyruvate in serum, brain cortex, and the liver using liquid chromatography–mass spectrometry (LC-MS) (Fig. 1A). We detected labeled pyruvate and lactate in the cortex, suggesting that these metabolites cross the blood-brain barrier to enter the interstitial fluid (Fig. 1C). We then investigated the metabolic fate of pyruvate in various tissues by quantifying ^13^C-labeled intermediates of pyruvate metabolism in gluconeogenesis and oxidative phosphorylation (i.e., the TCA cycle) (Fig. 1B). Consistent with the well-established role of the liver in gluconeogenesis, ^13^C labeling of the gluconeogenic intermediate, fructose bisposphate (FBP) was higher in the liver than the brain (Fig. 1C and table S1). In contrast, ^13^C labeling of intermediates of the TCA cycle, such as citrate, succinate, and malate, and their amino acid derivatives, glutamate and aspartate, was significantly more pronounced in the brain compared to serum and the liver indicating preferential oxidation of pyruvate in the brain (Fig. 1C and table S1). Together, our data demonstrate that pyruvate enters the brain from the circulation where it is efficiently broken down by oxidative phosphorylation. These findings indicate that pyruvate is a bona fide fuel source for the brain under physiological conditions.

Our in vivo metabolomics analysis captures the combined metabolic fates of pyruvate across all cell types comprising brain tissue, mainly neurons and glia. To examine pyruvate metabolism in a less heterogeneous population, we asked whether primary cultures of rat cortical neurons can use pyruvate as a metabolic fuel in the absence of glucose. In these cultures, glial expansion was inhibited after 2 days in vitro (DIV), and immunostaining for neuronal (NeuN) and astrocytic [glial fibrillary acidic protein (GFAP)] markers confirmed that the cultures were enriched for neurons (~65% of total cells) (fig. S1, A and B). The low abundance of glia restricts the metabolic activity and overall health of older cultures; therefore, experiments were performed after 7 to 8 DIV when neurons were undergoing synaptogenesis and remained healthy (*25*). To assess cellular viability under different metabolic conditions, cortical cultures were first established under standard conditions and at 7 to 8 DIV were divided into three groups: supplied with glucose (5 mM), supplied with an equimolar mixture of lactate and pyruvate (5 mM each) (denoted as lac/pyr) in the absence of glucose, or provided with neither glucose nor lac/pyr (i.e., fuel deprivation) for 12 to 16 hours (Fig. 1D). While fuel deprivation dramatically reduced cellular survival to ~20% of the glucose (control) condition, supplementation with lac/pyr in the absence of glucose restored survival to ~50% of the control without changing the relative proportion of neurons versus astrocytes (Fig. 1E and fig. S1B). This finding demonstrates that pyruvate supply can partially sustain the viability of cortical cultures even in the absence of glucose. To investigate pyruvate metabolism in more detail, primary cortical cultures supplied with glucose or pyruvate were analyzed with LC-MS–based targeted metabolomics. While cultures supplied with glucose released pyruvate into the culture media, those supplied with pyruvate were observed to take up extracellular pyruvate (Fig. 1F), supporting its potential use as an energy source. Measurement of steady-state levels of intracellular pyruvate and other TCA cycle intermediates with LC-MS revealed higher levels in cultures supplied with pyruvate, consistent with elevated flux of pyruvate through the TCA cycle (Fig. 1G and table S2). These findings indicate that neuronal cultures efficiently oxidize pyruvate as an alternative energy source to glucose, and neuronal pyruvate metabolism can partially sustain neuronal viability.

### Mitochondrial pyruvate uptake is essential for energy metabolism in nerve terminals

**Fig. 2.**
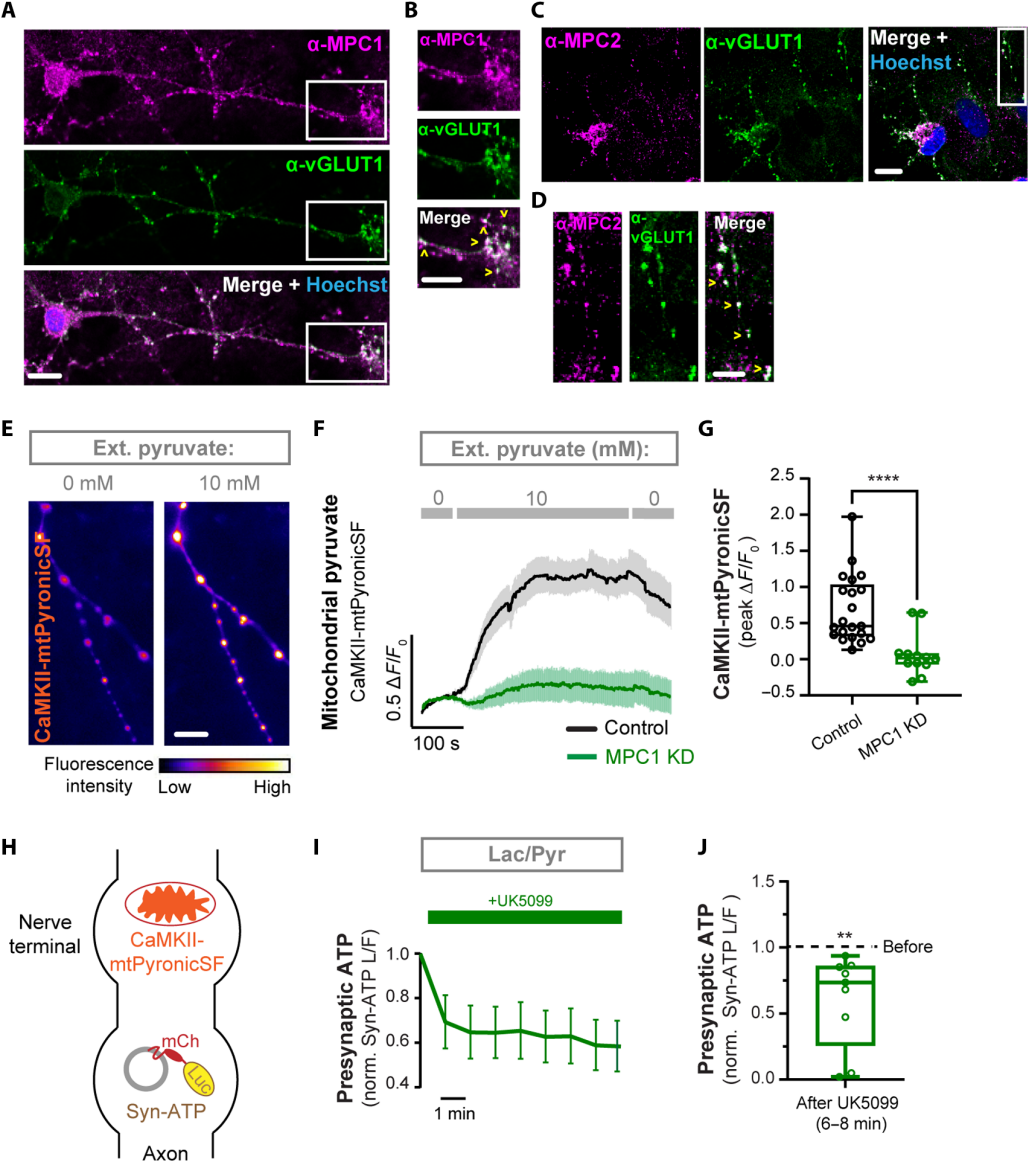
MPC is essential for pyruvate metabolism in nerve terminals. (**A** and **C**) Co-immunostaining of hippocampal neurons with antibodies against MPC1 (A) or MPC2 (C) and the presynaptic protein vGLUT1. Scale bars, 10 μm. (**B** and **D**) Magnification of the boxed areas in (A) and (C). Colocalization denoted with arrowheads. Scale bars, 10 μm (B) and 5 μm (D). (**E**) Representative images of axonal mitochondria expressing a mitochondrial pyruvate sensor, CamKII-mtPyronicSF, showing an increase in fluorescence intensity with perfusion of 10 mM extracellular pyruvate compared to 0 mM. Scale bar, 10 μm. (**F**) Average traces of CamKII-mtPyronicSF (Δ*F*/*F*_0_) showing inhibition of mitochondrial pyruvate uptake in neurons expressing shRNA against *mpc1* (MPC1 KD). F: fluorescence intensity. *n* = 12 to 18 (neurons). (**G**) Maximal CamKII-mtPyronicSF fluorescence response in 10 mM pyruvate as determined from traces in (F). (**H**) Schematic of the pyruvate sensor CaMKII-mtPyronicSF and the ATP indicator Syn-ATP expressed in nerve terminals. (**I**) Presynaptic ATP traces in terminals supplied with lactate and pyruvate (lac/pyr) after incubation with the MPC inhibitor UK5099 (100 μM), normalized to preincubation levels. *n* = 9 (neurons). (**J**) Presynaptic ATP level after application of MPC inhibitor UK5099 normalized to ATP level before. The box-whisker plots denote median (line), 25th to 75th percentile (box), and minimum-maximum (whiskers). Man-Whitney *U* test (G) and one sample *t* test (J). Error bars are SEM.

The MPC complex is expressed at high levels in the nervous system (*21*); however, little is known about its subcellular distribution in neurons. To determine whether MPC is present in nerve terminals where there is substantial energy demand for synaptic transmission, dissociated hippocampal neurons were immunostained with antibodies against MPC subunits (MPC1 and MPC2) and the presynaptic protein vesicular glutamate transporter 1 (vGLUT1). Both antibodies were previously validated for immunostaining in MPC1 (*21*, *22*) or MPC2 (*26*, *27*) knockout and knockdown (KD) tissues. We found that MPC1 and MPC2 subunits were present in neuronal cell bodies (Fig. 2, A and C), but they also colocalized with vGLUT1, indicating their expression in presynaptic terminals (Fig. 2, B and D).

To determine whether the MPC complex mediates mitochondrial pyruvate uptake in presynaptic mitochondria, we developed a quantitative optical assay for mitochondrial pyruvate uptake using the fluorescent pyruvate sensor PyronicSF. In this sensor, the bacterial transcription factor PdhR is fused with cyclically permuted green fluorescent protein (cpGFP) and targeted to the mitochondrial matrix with a COX8 mitochondrial targeting sequence (*23*). To improve the expression of PyronicSF in neurons, a construct using the CaMKII promoter (*28*) was developed, denoted as CaMKII-mtPyronicSF (Fig. 2H). In neuronal axons expressing the sensor, the perfusion of 10 mM extracellular pyruvate led to a significant increase in mitochondrial fluorescence (Fig. 2, E to G), consistent with the import of pyruvate into the mitochondrial matrix. The extracellular pyruvate concentration of 10 mM was selected on the basis of the affinity and dynamic range of the parental PyronicSF sensor (*23*). Depletion of neuronal MPC1 with short hairpin RNA (shRNA) (denoted as MPC1 KD) completely abolished CaMKII-mtPyronicSF fluorescence response to extracellular pyruvate, thus validating proper subcellular localization and specificity of the reporter (Fig. 2, F and G). This finding also indicates that the MPC complex is essential for pyruvate accumulation in axonal mitochondria. The efficiency of MPC1 depletion was confirmed by quantitative polymerase chain reaction (qPCR) of mpc1 mRNA in cortical neuronal cultures (fig. S2A). Because neuronal MPC2 protein was previously found to be unstable when MPC1 was depleted (*22*), MPC1 KD effectively ablates both components of the complex.

The presynaptic abundance of MPC subunits led us to investigate the effects of MPC inhibition on presynaptic ATP levels. The pharmacological inhibition of MPC with the compound UK5099 (*29*) in neurons expressing the genetically encoded ATP indicator Syn-ATP (*30*) (Fig. 2H) caused significant depletion of presynaptic ATP in the presence of an equimolar mixture of lactate and pyruvate, indicating the critical role of MPC in pyruvate oxidation (Fig. 2, I and J). We confirmed that presynaptic ATP level was not affected by UK5099 treatment when neurons were supplied with glucose (fig. S2B). Together, these results demonstrate that the MPC complex is present in presynaptic mitochondria and is essential for oxidative pyruvate metabolism in nerve terminals.

### Mitochondrial pyruvate uptake regulates distinct steps in the SV cycle

**Fig. 3.**
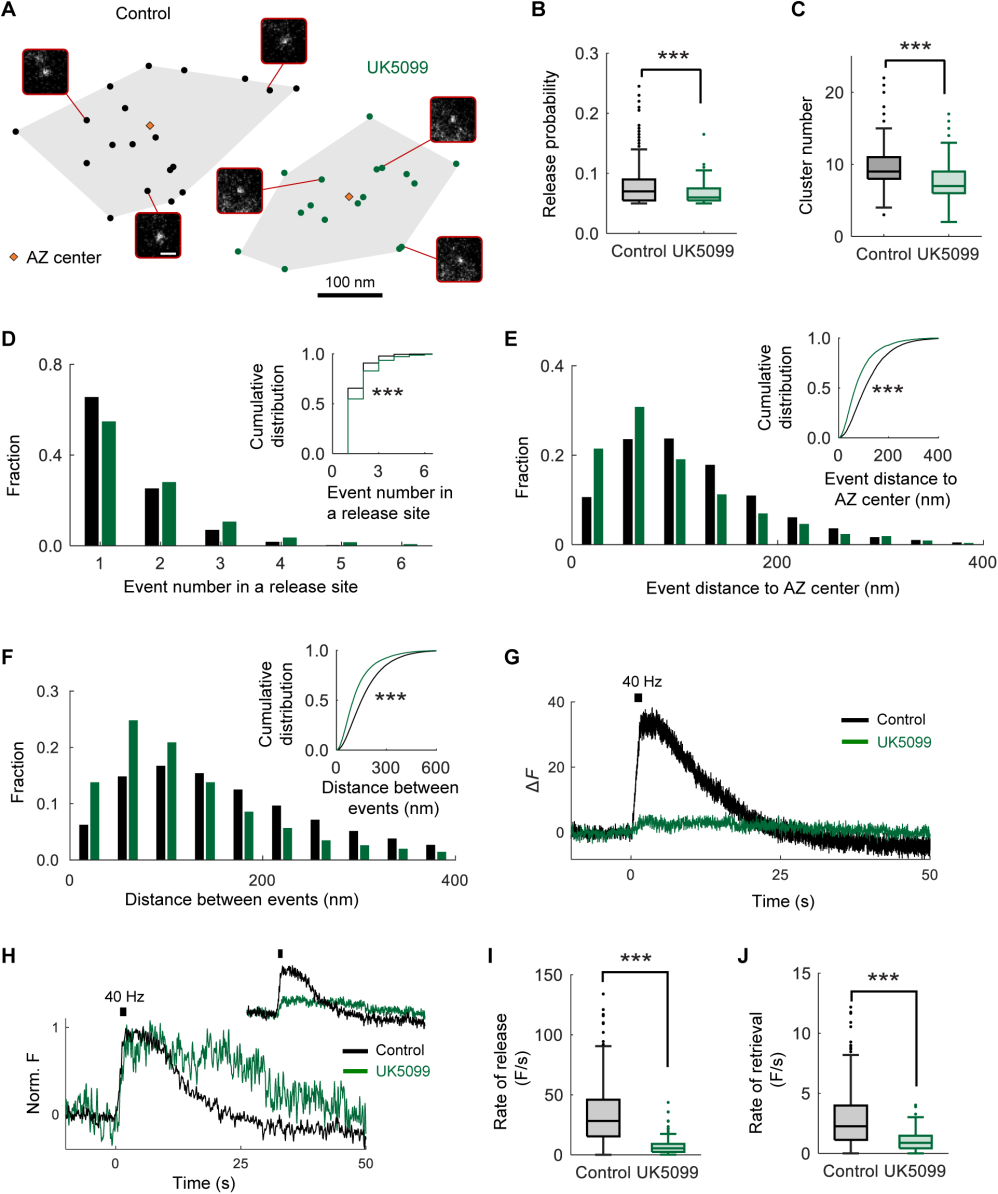
Mitochondrial pyruvate uptake regulates the spatiotemporal properties of SV release and retrieval. (**A**) Two examples of AZs with the localization of release events without (left) or with (right) UK5099. Representative images of individual events evoked at 1 Hz are shown for each AZ. (**B**) Average release Pr of events evoked by 1-Hz stimulation for 200 s in the two conditions. Sample number (cultures/coverslips/synapses): Control: 3/16/691, UK5099: 3/9/230. (**C**) Average number of clusters (release sites) in an individual AZ. Sample number: Control: 3/16/732, UK5099: 3/9/220. (**D**) Number of release events detected per release site in the two conditions (plotted as fraction of total events). The inset shows the cumulative distribution plot. Sample number (cultures/coverslips/release sites): Control: 3/16/7005, UK5099: 3/9/1705 for cultures/coverslips/synapses/release sites. (**E**) Distribution of distances from release events to the center of the AZ (plotted as fraction of total events). The inset shows the cumulative distribution plot. Sample number (cultures/coverslips/release sites): Control: 3/16/9097, UK5099: 3/9/2786 for cultures/dishes/events. (**F**) Distribution of distances between consecutive events over time (plotted as fraction of total events). The inset shows the cumulative distribution plot. Sample number (cultures/coverslips/events): Control: 3/16/9097, UK5099: 3/9/2786 for cultures/dishes/events. (**G** and **H**) Average (G), normalized (H), and raw [(H) inset] representative traces of vesicle release evoked by high-frequency stimulation (50 AP at 40 Hz) in the two conditions. a.u., arbitrary units. (**I** and **J**) Rates of vesicle exocytosis (I) and endocytosis (J) in the two conditions calculated by linear fitting of the rise and decay components of the train responses during high-frequency stimulation. Sample number (cultures/coverslips/synapses): Control: 3/16/691, UK5099: 3/9/230. All experiments were performed in the presence of lactate and pyruvate (no glucose). Kolmogorov-Smirnov test [(B) to (F), (I), and (J)]. Error bars are SEM. **P < 0.001.

The release and subsequent recycling of SVs, collectively known as the SV cycle, is a critical process in neurotransmission. Multiple steps in the SV cycle including SV docking, release, and the replenishment of the readily releasable pool (*31*–*33*) have been shown to be ATP-dependent and highly susceptible to metabolic perturbations, such as mitochondrial or glycolytic inhibition (*12*, *30*, *34*, *35*). Because our results revealed that mitochondrial pyruvate uptake via MPC is essential for oxidative ATP synthesis in nerve terminals (Fig. 2), we investigated whether MPC function regulates specific steps in the SV cycle by altering ATP availability. To examine SV release at a single-vesicle level in individual hippocampal synapses in culture, we used our previously established near–total internal reflection fluorescence (TIRF) imaging approach combined with well-established computational detection and localization methods (*35*–*37*). We visualized vesicle release with vGLUT1-pHluorin (*38*), which contains a pH-sensitive indicator pHluorin targeted to the SV lumen via fusion with vGLUT1. Neurons were acutely supplied with an equimolar mixture of lactate and pyruvate (denoted as lac/pyr) replacing glucose to enhance metabolic reliance on mitochondrial OXPHOS as we previously reported (*12*). Individual release events were evoked by single action potential (AP) stimulation at 1 Hz for 200 s and then automatically detected and localized within the synaptic active zone (AZ) with ~27-nm precision using computational detection and localization approaches, as previously described (*36*, *37*, *39*). The functional AZ area was defined as a convoluted hull encompassing all events identified within an individual synapse, with the centroid defining the AZ center (Fig. 3A). This definition of the AZ area is consistent with AZ dimensions observed in ultrastructural studies by electron microscopy (*39*, *40*). The detected SV release events were clustered to define the location of individual release sites within each AZ using a hierarchical clustering algorithm with a clustering diameter of 50 nm (*39*–*41*).

Using this approach, we first determined whether MPC inhibition affected basal vesicle release probability (Pr). Acute pharmacological inhibition of MPC with UK5099 significantly reduced the Pr measured at 1-Hz stimulation (Fig. 3B). SV release is not randomly distributed across the AZ but is characterized by a repeated utilization of several specialized release sites within the AZ (*36*, *37*, *40*–*43*). Given the reduction in Pr and the energetic dependencies of SV release, we next asked whether the spatial organization of SV release is affected by MPC inhibition. We found that the number of vesicle release sites, denoted as cluster number, was significantly reduced (Fig. 3C) while repeated utilization of the same release sites increased with MPC inhibition (Fig. 3D). Furthermore, SV release events occurred at notably shorter distances from the AZ center (Fig. 3E), and the distance between events was also substantially reduced when MPC was inhibited (Fig. 3F). These results suggest that mitochondrial pyruvate uptake during single AP firing regulates the spatial dynamics of SV release shifting release toward a subset of more centrally located sites, as depicted schematically (Fig. 3A).

The energetic demands of synaptic transmission scale with the duration and intensity of stimulation (*34*). Therefore, we examined the impact of MPC inhibition on SV release and retrieval evoked by high-frequency stimulus trains of 50 AP at 40 Hz in hippocampal terminals supplied with lactate and pyruvate replacing glucose (Fig. 3, G to J). We found that SV release was substantially impaired when MPC was acutely inhibited with UK5099 (Fig. 3, G and I). The retrieval of SVs after release is also known to be highly sensitive to energetic perturbations (*12*, *14*, *30*). MPC inhibition markedly slowed SV retrieval following stimulation by a train of 50 AP at 40 Hz (Fig. 3, H and J). We confirmed that SV retrieval kinetics was similarly reduced following a longer stimulation paradigm (100 AP at 10 Hz) with application of UK5099 (fig. S3, A and B) or Zaprinast, another pharmacological inhibitor of MPC (fig. S3, C and D) (*44*). Together, our data demonstrate that mitochondrial pyruvate transport by the MPC complex regulates the spatiotemporal properties of SV release and retrieval in nerve terminals, both during single AP firing and high-frequency trains.

### Sirtuin 3 modulates mitochondrial pyruvate uptake and acetylation of MPC complex

Lysine acetylation is a reversible posttranslational modification that plays a major role in modulation of mitochondrial function (*45*). While it is thought that mitochondrial proteins are acetylated nonenzymatically, the removal of acetyl groups is catalyzed by mitochondrial sirtuins, primarily Sirtuin 3 (Sirt3), a nicotinamide adenine dinucleotide–dependent deacetylase. We previously discovered that neuronal glucose deprivation induces Sirt3 expression, which, in turn, stimulates oxidative metabolism in nerve terminals (*46*). Hyperacetylation of MPC2 has been reported in the liver of *Sirt3^−/−^* mice (*47*) and in diabetic heart (*48*), implicating acetylation as a potential mechanism for modulation of MPC activity.

**Fig. 4.**
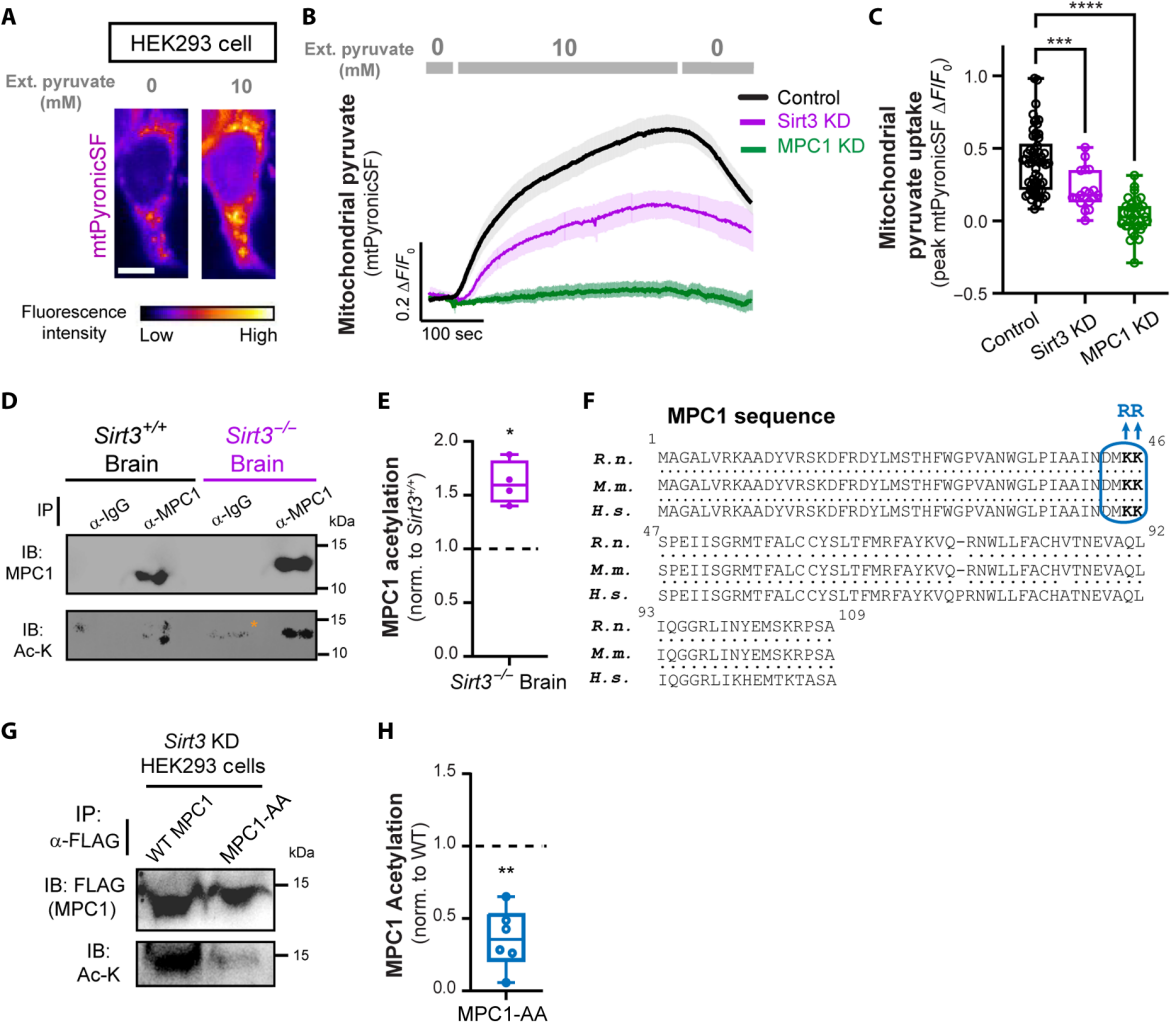
Sirt3 modulates mitochondrial pyruvate uptake and MPC acetylation. (**A**) Representative images of an HEK293 cell expressing mtPyronicSF in media containing 0 and 10 mM pyruvate. (**B**) Average traces of mtPyronicSF showing differential mitochondrial pyruvate accumulation in control HEK293 cells and cells expressing shRNA against sirt3 (Sirt3 KD) or mpc1 (MPC1 KD). (**C**) Peak values of mtPyronicSF (Δ*F*/*F*_0_) in response to 10 mM pyruvate, as determined from traces in (B). *n* = 24 to 57 fields of view (FOV), five to six cells per FOV. (**D**) Immunoprecipitation (IP) of endogenous MPC1 from *Sirt3^+/+^*and *Sirt3^−/−^* mouse brain lysates with antibodies against MPC1 and rabbit immunoglobulin G (IgG) (control) followed by immunoblotted (IB) with antibodies against MPC1 (top) and Ac-K (bottom). Asterix denotes a nonspecific band in the IgG immunoprecipitate. (**E**) Intensity of Ac-K band normalized to MPC1 band from (D), expressed relative to *Sirt3^+/+^* control. *n* = 4 (IP blots). Raw intensities of Ac-K normalized to MPC1 in *Sirt3^+/+^*and *Sirt3^−/−^* brains were used for statistical comparison. (**F**) Alignment of rat (*Rattus norvegicus, R. n.*), mouse (*Mus musculus, M. m.*), and human (*Homo sapiens, H. s.*) MPC1 protein sequences showing conservation of an acetylation motif (boxed in blue). A nonacetylatable form was constructed by mutation of K45/46 to A45/A46. (**G**) Immunoprecipitation of wild-type (WT) MPC1 or acetyl mutant (MPC1-AA) from Sirt3-deficient (KD) HEK293 cells, immunoblotted for FLAG (top) and Ac-K (bottom). (**H**) Intensity of Ac-K band normalized to FLAG intensity from panel G, expressed relative to WT-MPC1. *n* = 6 (IP blots). Kruskal-Wallis test (C), paired *t* test (E), and one sample *t* test (H). Error bars are SEM. IB, immunoblot. *P < 0.05; **P < 0.01; ***P < 0.001; ****P < 0.0001.

These previous findings prompted us to ask whether deacetylation of mitochondrial proteins by Sirt3 regulates the pyruvate transport activity of the MPC complex. To examine mitochondrial pyruvate uptake, the optical pyruvate sensor mtPyronicSF was expressed in human embryonic kidney (HEK) 293 cells, and extracellular pyruvate concentration was changed from 0 to 10 mM, triggering a reversible rise in the fluorescence intensity of mtPyronicSF consistent with mitochondrial pyruvate accumulation (Fig. 4, A to C). Similar to neuronal axons (Fig. 2, E to G), this response was fully blocked by shRNA-mediated depletion of MPC1 (MPC1 KD) (Fig. 4, B and C) which was confirmed by qPCR of mpc1 mRNA (fig. S4A). The shRNA-mediated Sirt3 KD significantly blunted mitochondrial pyruvate accumulation, implicating Sirt3 in modulation of mitochondrial pyruvate transport (Fig. 4, B and C). The efficiency of Sirt3 KD was confirmed by Western blotting for Sirt3 protein (fig. S4, B and C). Because the proton gradient across the inner mitochondrial membrane provides the driving force for pyruvate transport by the MPC complex (*49*), we confirmed that MPC1 KD or Sirt3 KD did not alter mitochondrial membrane potential in HEK293 cells using the mitochondrial membrane dye tetramethylrhodamine methyl ester (TMRM; fig. S4D).

Our data suggest that Sirt3 regulates pyruvate entry into the mitochondrial matrix, potentially through posttranslational modification of MPC subunits. To further examine this possibility, we investigated whether Sirt3 abundance affects the acetylation state of the MPC complex in the brain. We focused on MPC1 because, unlike MPC2, acetylation of this subunit has not been reported in proteomic studies and remains poorly explored (*48*). MPC1 was immunoprecipitated from *Sirt3^+/+^* (*Sirt3^fl/fl^*) and *Sirt3^−/−^* mouse (*50*) brain tissues, and the immunoprecipition (IP) fraction was immunoblotted with a pan-acetyl-lysine antibody, revealing the hyperacetylation of MPC1 in *Sirt3^−/−^* brain as compared to *Sirt3^+/+^* tissue (Fig. 4, D and E). The specificity of MPC1 IP band was confirmed with an immunoglobulin G (IgG) antibody. Our results thus demonstrate that MPC1 is a substrate for Sirt3-mediated deacetylation in the brain.

To further confirm MPC1 acetylation, we sought to map the acetylated lysine residues. Bioinformatics analysis of lysine acetylation in mitochondrial proteins from rat brain and brown fat has identified a consensus sequence motif for lysine acetylation (D-X-X-AcK) (*51*), which is conserved in MPC1 sequences from rats, mice, and humans (Fig. 4F, boxed in blue). To confirm if the conserved lysines in MPC1 were indeed acetylated as predicted, we constructed a FLAG-tagged MPC1 in which K45 and K46 motif sites were replaced with alanine (denoted as MPC1-AA), blocking acetylation. To detect MPC1 acetylation, FLAG-tagged wild-type (WT) MPC1 or MPC1-AA were expressed and immunoprecipitated from Sirt3-deficient HEK293 cells in which mitochondrial proteins are known to be hyperacetylated (*52*, *53*). Sirt3 KD with CRISPR-Cas9 gene editing was confirmed with immunoblotting HEK293 lysates from successive passages with an antibody against Sirt3 (fig. S4E). While both MPC1-WT and MPC1-AA were pulled down with anti-FLAG antibody, immunoblotting the IP fraction with a pan-acetyl-lysine antibody revealed significant reduction in acetylation of MPC1-AA as compared to WT MPC1 (Fig. 4, G and H). This finding confirms K45 and K46 as the primary acetylated residues in MPC1. Together, our results suggest that Sirt3 regulates MPC1 acetylation in the brain, and Sirt3 depletion impairs mitochondrial uptake of pyruvate.

### An acetyl mimetic MPC1 mutant impairs mitochondrial pyruvate uptake and synaptic transmission

**Fig. 5.**
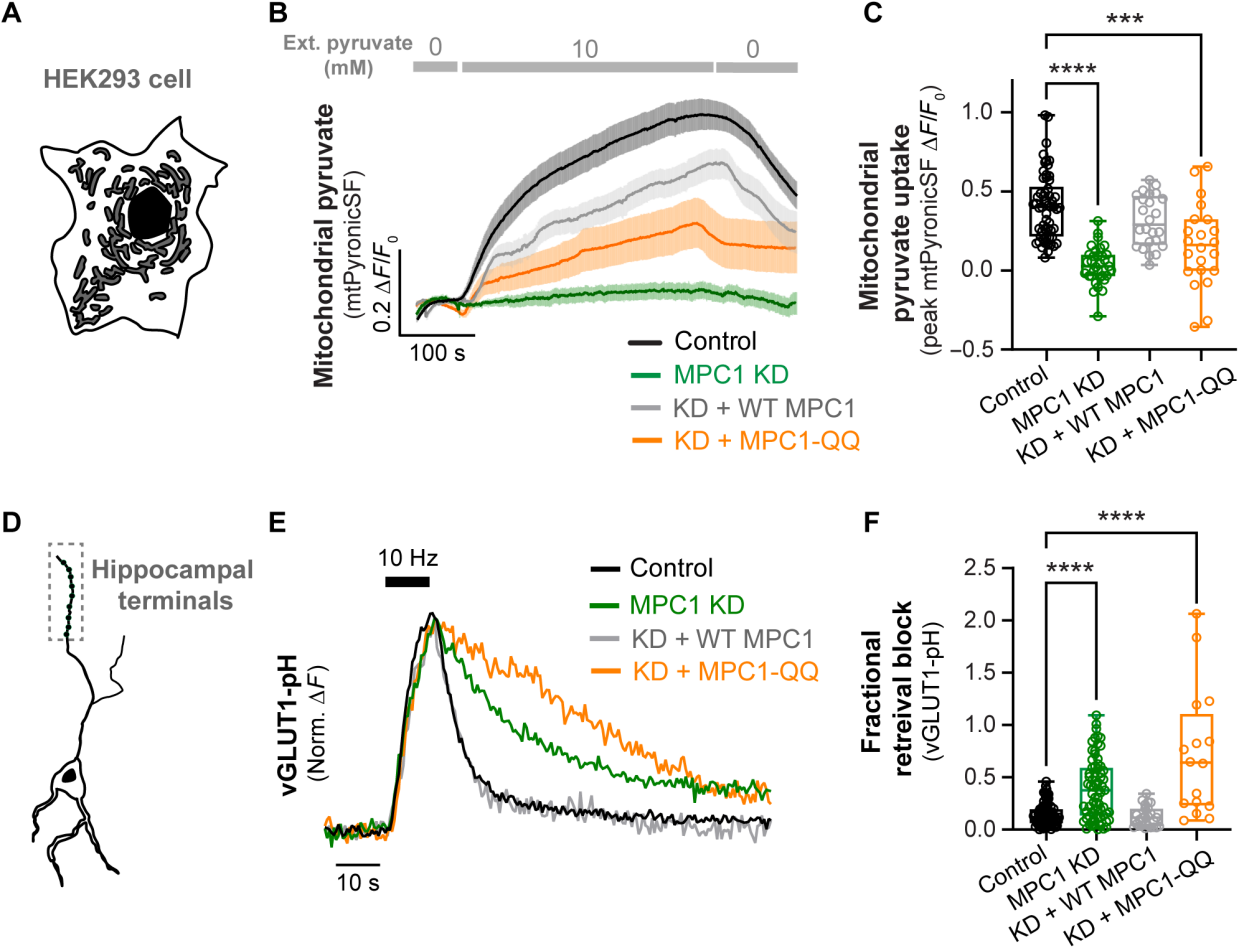
An acetyl mimetic MPC1 mutant impairs mitochondrial pyruvate uptake and SV retrieval. (**A**) Schematic of a HEK293 cell expressing mtPyronicSF. (**B**) Average traces of mtPyronicSF expressed in HEK293 cells showing differential mitochondrial pyruvate accumulation in control, MPC1 KD, MPC1 KD + WT MPC1, or MPC1 KD + MPC1-QQ (acetyl mimetic mutant). Control and MPC1 KD traces are the same as in Fig. 4B. (**C**) Peak values of mtPyronicSF (Δ*F*/*F*_0_) in response to perfusion of 10 mM pyruvate, as determined from traces in (B). *n* = 22 to 57 FOV, five to six cells per FOV. (**D**) Schematic of a hippocampal neuron-expressing vGLUT1-pH. (**E**) Sample normalized vGLUT1-pH traces in hippocampal terminals electrically stimulated with 100 AP at 10 Hz in control neurons, or neurons expressing MPC1 KD, MPC1 KD + WT MPC1, or MPC1 KD + MPC1-QQ (acetyl mimetic mutant). (**F**) SV retrieval quantified as fractional retrieval block calculated from traces in (E). *n* = 16 to 81 (neurons). Black bar denotes electrical stimulation. Kruskal-Wallis test [(C) and (F)]. Error bars are SEM. ***P < 0.001; ****P < 0.0001.

The modulation of MPC1 acetylation by Sirt3 prompted us to investigate whether this posttranslational modification regulates the pyruvate transport activity of the MPC complex. To determine whether acetylated MPC1 alters mitochondrial pyruvate transport, we constructed an MPC1 mutant in which the two acetylated lysine residues (K45 and K46) were mutated to glutamine (denoted as MPC1-QQ) because glutamines are effective mimetics of lysine acetylation (*54*). We confirmed that both WT MPC1 and MPC1-QQ were expressed in HEK293 cells (fig. S5). We then assessed how the expression of these MPC1 constructs in MPC1-deficient HEK293 cells affects mitochondrial pyruvate uptake using the optical sensor mtPyronicSF. As shown before (Fig. 4C), mtPyronicSF fluorescence in MPC1 KD cells did not change in response to increasing extracellular pyruvate concentration from 0 to 10 mM; however, this defect could be rescued with the expression of WT MPC1 (Fig. 5, A to C). In contrast, the expression of MPC1-QQ in MPC1 KD cells failed to restore mitochondrial pyruvate uptake (Fig. 5, B and C). This result indicates that acetyl mimetic MPC1 mutant is defective in pyruvate transport, consistent with the idea that acetylation of the MPC complex impairs its activity. Given the critical role of MPC in regulating the SV cycle in hippocampal nerve terminals (Fig. 3), we then examined the effects of MPC1 acetylation on SV retrieval following electrical stimulation using the optical reporter vGLUT1-pH. Consistent with pharmacological inhibition of MPC1 (Fig. 3 G to I, and fig. S3), SV retrieval following a stimulus train of 100 AP at 10 Hz was significantly slower in MPC1 KD terminals compared to the control (Fig. 5, D to F). While this defect was fully reversed with expression of WT shRNA-resistant MPC1, acetyl-mimetic MPC1-QQ failed to rescue SV retrieval in MPC1 KD terminals (Fig. 5, E and F), consistent with its inability to facilitate mitochondrial pyruvate transport (Fig. 5, A to C). Together, our data reveal that acetylated MPC1 is deficient in mitochondrial pyruvate transport and metabolic support of synaptic transmission, thus establishing the functional significance of lysine acetylation in the regulation of MPC complex.

## DISCUSSION

Glucose has long been considered the canonical energy source for the brain. Yet, glucose availability undergoes spatial and temporal fluctuations in active brain regions, during sleep and fasting. Thus, the mammalian brain may have evolved to scavenge for alternative fuels such as pyruvate to protect against loss of cognitive function and ensure organismal survival in the face of unpredictable food supply. The remarkable capacity of the brain for pyruvate oxidation compared to other tissues may be explained by its steep energetic demands and the catastrophic consequences of brain energetic failure. Here, using in vivo metabolomics and isotope tracing, we demonstrate that circulating blood delivers pyruvate to the brain where it is efficiently broken down by oxidative phosphorylation. We found that neuronal cultures can use pyruvate in the absence of glucose, and pyruvate metabolism in nerve terminals relies on mitochondrial pyruvate uptake by the MPC complex. MPC activity in turn regulates the spatiotemporal properties of evoked SV release and retrieval. In addition, mitochondrial pyruvate uptake is modulated by lysine acetylation of MPC1 subunit, which impairs its transport function.

Along with serum pyruvate, lactate is also produced locally by astrocytes from the glycolytic breakdown of glucose and provided to neurons in a process known as the astrocyte-neuron lactate shuttle model (*55*). The existence of multiple routes for the delivery of pyruvate or lactate to neurons underscores the importance of this fuel type for neuronal metabolism. However, further work is needed to delineate the physiological roles of local versus systemic delivery of pyruvate to the brain, and their respective contributions to supporting neuronal function during dietary restrictions or intense circuit activity.

A major fraction of brain energy is directed to nerve terminals to support synaptic transmission. Here, we identified MPC-dependent pyruvate entry into the TCA cycle as a critical node in the metabolic regulation of the SV cycle. We showed that mitochondrial pyruvate transport is crucial for the spatiotemporal control of SV release and subsequent SV retrieval in the absence of glucose. Inhibition of mitochondrial pyruvate uptake not only reduced SV release probability but also shifted release closer to AZ center. This may be due to the lower energetic barrier for SV release in the center than the periphery of AZ, which is consistent with our earlier studies where we found that release sites at the AZ center have higher rates of utilization and release Pr (*39*). Although it is not known why the energetic barrier for SV release is lower near the AZ center, SV release may require fewer ATP molecules at the center where nanoclusters of molecular components of docking and release machinery are larger and more stable as compared to the periphery where these components are more mobile (*56*–*58*). As with release, SV retrieval is also known to be energetically demanding (*14*, *30*), requiring ATP for the pinching of vesicles, clathrin uncoating, vesicle refilling, and the replenishment of the readily releasable pool (*31*, *32*). We found that MPC inhibition effectively blocked SV retrieval during high-frequency stimulation, indicating that MPC function is indispensable for metabolic support of the SV cycle under oxidative conditions.

Here, we showed that the mitochondrial deacetylase Sirt3 modulates MPC acetylation and its pyruvate transport function. The energetic needs of nerve terminals are highly variable, and the regulation of pyruvate entry to the mitochondrial matrix via posttranslational acetylation of MPC may serve as a molecular rheostat matching oxidative ATP synthesis with presynaptic energy demand. We previously showed that Sirt3 deacetylates a number of mitochondrial proteins during neuronal glucose deprivation. Therefore, we hypothesize that changes in Sirt3 expression in response to physiological stimuli—such as glucose deprivation (*46*), prolonged synaptic activity (*59*), or in the pathological conditions of diabetes (*60*) and neurodegeneration (*61*)—may ultimately regulate neuronal pyruvate metabolism through MPC acetylation. Considering the critical role of MPC in modulation of synaptic transmission, it will be important for future studies to determine the effects of MPC acetylation on synaptic plasticity and cognitive performance under these conditions.

In the present study, we mapped two lysine acetylation sites (K45 and K46) in MPC1 and identified the functional significance of these modifications for mitochondrial pyruvate uptake. One limitation of the present study is our lack of information regarding how lysine acetylation affects the structural conformation of MPC1 to regulate pyruvate transport. While atomic structures of MPC subunits and the assembled complex are not available, structural predictions by AlphaFold localize K45 and K46 to a flexible loop connecting two transmembrane helices. Further structure-function studies are needed to delineate the significance of this loop in pyruvate transport. Although we did not directly examine MPC2 in this study, hyperacetylation of MPC2 has been observed in diabetic mouse heart (*48*). Similar to MPC1 mutants, acetyl mimetic mutations of MPC2 were shown to dampen oxidative metabolism in cardiac muscle, presumably by reducing mitochondrial pyruvate uptake (*48*). Because MPC1 and MPC2 are essential components of the same of transport complex in neurons and other cell types, we anticipate that both subunits are similarly regulated by acetylation. Beyond lysine acetylation, there is also growing evidence for the reprogramming of oxidative metabolism by other forms of posttranslational modifications of mitochondrial proteins, such as phosphorylation (*62*), O-GlcNAcylation (*63*), and succinylation (*64*). Therefore, it is important to examine the regulation of neuronal MPC by these modifications and determine their physiological role in the metabolic plasticity of synaptic transmission.

Pyruvate has been shown to be critical for memory formation (*65*), particularly when local glucose levels decline in active circuits during memory tasks (*5*). Furthermore, the activity of pyruvate dehydrogenase, which catalyzes the first step in pyruvate oxidation, strongly correlates with the intensity of neuronal firing (*66*). However, an important unresolved question is to what extent pyruvate metabolism varies across different brain regions, such as the hippocampus, cerebellum, or cortex, and whether these regions differ in their metabolic plasticity in usage of fuel types, such as glucose, pyruvate, or ketone bodies. Understanding the metabolic plasticity of brain function in molecular detail has profound clinical implications for human physiology. Metabolic reprograming through MPC inhibition has emerged as an insulin-sensitizing strategy for treatment of type 2 diabetes and has been shown to attenuate neurodegeneration in certain Parkinson’s disease models (*19*, *20*). Our work implicates mitochondrial pyruvate transport in the regulation of synaptic transmission, highlighting the clinical importance of elucidating the effects of MPC-modulating drugs on cognitive function in healthy individuals and patients suffering from metabolic and neurodegenerative diseases.

## MATERIALS AND METHODS

### Experimental design and subject details

#### Animals

Animal-related experiments were performed in accordance with protocols approved by the Washington University Institutional Animal Care and Use Committee (approval numbers 22-0391 and 22-0304). The following animal strains were used: WT rats of the Sprague-Dawley strain (RRID: RGD_734476), 129S6 WT and Sirt3^tm1.1Cxd^/Sirt3^tm1.1Cxd^ mice (*50*), and male C57BL/6 for pyruvate tracing experiments.

#### Primary neuronal cultures

Hippocampi were dissected from 1- to 3-day old (mixed sex) Sprague-Dawley rat pups and dissociated. Mixed cultures of neurons and glia were plated on coverslips coated with poly-ornithine and after 6 to 8 days of plating and were transfected with calcium phosphate, as previously described (*46*, *67*). Hippocampal neurons were cultured in media containing 0.6% glucose, bovine transferrin (0.1 g/liter; MilliporeSigma, 616420), insulin (0.25 g/liter), GlutaMAX supplement (0.3 g/liter; Thermo Fisher Scientific, 35050-061), 2% N-21 (R&D Systems, AR008), 5% fetal bovine serum (FBS) (R&D Systems, S11510). Cytosine β-d-arabinofuranoside (Ara-C) was added at 2 to 3 DIV to limit glial proliferation. Cortical neurons were maintained in culture media containing Neurobasal-A (NB-A) (Gibco, #A24775-01) supplemented with 2% N-21, 5% FBS, and other nutrients. Cultures were incubated at 37°C in a 95% air and 5% CO_2_ humidified incubator, and experiments were performed at 14 to 21 DIV (hippocampal neurons) or 8 to 14 DIV (cortical neurons).

#### HEK293 cell cultures

Cells were maintained in culture media composed of Dulbecco’s modified Eagle’s medium (Gibco, 11966-025), 10% FBS (Gibco, 26140-079), and penicillin/streptomycin (10,000 U/ml) at 37°C in a 95% air and 5% CO_2_ humidified incubator. Cells were transfected when 30 to 40% confluent by calcium phosphate method or Lipofectamine 2000 (Thermo Fisher Scientific, 11668019).

### Method details

#### Plasmid constructs

All the plasmid constructs and key reagents used here are listed in Table 1. The following previously published DNA constructs were used: Syn-ATP (*30*), vGLUT1-pHluorin (*38*), mtPyronicSF [Addgene, plasmid 124813; (*23*)], and plenti.CamKII.(synapto).iATPSnFR2.A95A.A119L.miRFP670nano3 (*68*). CaMKII-mtpyronicSF construct was generated through insertion of mtPyronicSF into the Bam HI and Hind III sites of CaMKII-GCaMP6f.WPRE.SV40 (Addgene, plasmid 100834). To construct WT shRNA-resistant MPC1, mouse MPC1 sequence was the fused to a C-terminal FLAG tag, and three silent mutations were introduced in the regions complementary to rat shRNA. WT shRNA-resistant MPC1, MPC1-A_45_A_46_, and MPC1-Q_45_Q_46_ were codon-optimized for expression in rat cells using the GeneOptimizer tool (Thermo Fisher Scientific), synthesized (GeneArt Gene Synthesis, Thermo Fisher Scientific) and cloned into the Bam HI and Eco RI sites of pcDNA3.0. pLKO.1 [Addgene, plasmid 10878; (*69*)] and pLKO.1-TRCmTagBFP2 (Addgene, plasmid 191566) vectors were used for expression of shRNAs against *mpc1* (rat target sequence: CAAACGAAGTCGCTCAGCTCA, human target sequence: GCCTCGGAACTGGCTTCTGT) and *sirt3* (human target sequence:GTGGGTGCTTCAAGTGTTGTT).

**Table 1.** Key resource table. ATCC, American Type Culture Collection; IgG, immunoglobulin G; N/A, not applicable.

Reagent or Resource	Source	Identifier
Antibodies		
Anti-vGLUT1	MilliporeSigma	Catalog no. AB5905; RRID: AB_2301751
Anti-ATPase5β antibody	Sigma-Aldrich	Catalog no. HPA001520; RRID: AB_1078243
Anti-Sirt3 antibody	Cell Signaling Technology	Catalog no. 5490S; RRID: AB_10828246
Anti–acetylated lysine antibody	Cell Signaling Technology	Catalog no. 9441S; RRID: AB_331805
Anti-Flag antibody (IP)	Sigma-Aldrich	Catalog no. F1804; RRID: AB_262044
Anti-Flag antibody (Western blot)	Thermo Fisher Scientific	Catalog no. MA1-91878; RRID: AB_1957945
Normal rabbit IgG	MilliporeSigma	Catalog no. 12-370; RRID: AB_145841
Anti-MPC2 antibody	Thermo Fisher Scientific	Catalog no. 20049-1-AP; RRID; AB_10665371
Anti-MPC1 antibody (ICC)	MilliporeSigma	Catalog no. HPA045119; RRID: AB_10960421
Anti-MPC1 antibody (Western blot)	gift of B. Finck	N/A
Anti-GFAP antibody	Thermo Fisher Scientific	Catalog no. NB300141; RRID: AB_10001722
Anti-NeuN antibody	Thermo Fisher Scientific	MA5–33103; RRID: AB_2802653
Biological samples		
rMPC1 shRNA Lentivirus	Hope Center Viral Vectors Core at Washington University in St. Louis	N/A
pFU-vGluT1-pHGFP-W	Hope Center Viral Vectors Core at Washington University in St. Louis	N/A
Chemicals, peptides, and recombinant proteins		
Deacetylation inhibition cocktail	Santa Cruz Biotechnology	Catalog no. sc-362323
6-cyano-7nitroquinoxalibe-2, 3-dione (CNQX)	Sigma-Aldrich	Catalog no. C239-25MG
d,l-2-amino-5-phosphonovaleric acid (APV)	Sigma-Aldrich	Catalog no. A5282
Protein A agarose beads	Cell Signaling Technology	Catalog no. 9863
Anti-FLAG M2 magnetic beads	Sigma-Aldrich	Catalog no. M8823
U^13^C-Pyruvate	Cambridge Isotope Laboratories	CLM-2440-PK
Alt-R S.p. Cas9 Nuclease V3	IDT	1081058
UK5099	Sigma-Aldrich	Catalog no. 5048170001
Zaprinast	Sigma-Aldrich	Catalog no. Z0878-25MG
Critical commercial assays		
All-In-One 5X RT Master Mix	Applied Biosystems	Catalog no. ABM-G592
PowerUp SYB Green Master Mix	Thermo Fisher Scientific	Catalog no. A25742
TaqMan Fast Advanced Master Mix	Applied Biosytems	Catalog no. 4444557
Experimental models: Cell lines		
HEK293 cell line	ATCC	ATCC code: CRL-1573 RRID: CVCL_0045
Experimental models: Organisms/strains		
Sprague-Dawley Rat	Charles River Laboratories	Strain code: CD IGS SD # 001 RRID: RGD_734476
Sirt3^tm1.1Cxd^/Sirt3^tm1.1Cxd^	(*50*)	RRID: MGI:3822546
Recombinant DNA		
vGLUT1-pHluorin	(*38*)	N/A
Syn-ATP	(*30*)	N/A
plenti.CamKII.(synapto).iATPSnFR2.A95A.A119L.miRFP670nano3	(*68*)	Addgene #209730
CamKII-mtPyronicSF	This paper	
mtPyronic	(*23*)	Addgene #124813
pLKO.1 vector	(*69*)	Addgene #10878
pLKO.1 - TRC mTagBFP2	gift from T.A. Ryan	Addgene #191566
pLKO.1-hSirt3 shRNA	This paper	N/A
pLKO.1-hMPC1 shRNA	This paper	N/A
pLKO.1-mTagBFP2-rMPC1 shRNA	This paper	N/A
wt MPC1-FLAG	This paper	N/A
MPC1-AA-FLAG	This paper	N/A
MPC1-QQ-FLAG	This paper	N/A
Sequence-based reagents		
**qPCR primers**		**Sequence 5′-3′**
Rat *mpc1* forward	Thermo Fisher Scientific	AAACTGGCTTCTGTTTGCGTG
Rat *mpc1* reverse	Thermo Fisher Scientific	CCGCTTACTCATCTCGTAGTT
Human *Mpc1* TaqMan Probe	Thermo Fisher Scientific	Catalog no. Hs00211484_m1
Human *gapdh* TaqMan Probe	Thermo Fisher Scientific	Catalog no. Hs03929097_g1
Rat β*-actin* forward primer	Thermo Fisher Scientific	GTCGAGTCCGCGTCCAC
Rat β*-actin* reverse primer	Thermo Fisher Scientific	TATCGTCATCCATGGCGAACTGG
hSirt3 CRISPR-Cas9 sgRNA	IDT	CTCTACACGCAGAACATCGA
Software and algorithms		
ImageJ	National Institute of Health	RRID: SCR_003070
GraphPad Prism 9.2.0	GraphPad Software	RRID:SCR_002798
Jupyter Notebook	Project Jupyter	RRID:SCR_018315
Adobe Illustrator	Adobe Inc.	RRID:SCR_010279
MATLAB	MathWorks	RRID: SCR_001622

#### MTT survival assay and quantification of neurons and astrocytes

Cells were plated at a density of 1 × 10^5^ cells per well in precoated 96-well plates and at 2 DIV were fed with NB-A containing Ara-C to inhibit glial expansion. At 8 DIV, medium was replaced for with medium containing either glucose (5 mM) or mixture of lactate and pyruvate (5 mM each) or starvation media (only NB-A) and placed in a 5% CO_2_ incubator at 37°C for 16 hours. MTT solution [Cell proliferation kit I (MTT), Roche, 11465007001) was then added to each well for 4 hours, followed by incubation with a solubilizing solution overnight. Optical density was measured at 450 nm according to the manufacturer’s instructions, and background signal (in media-only wells) was subtracted from every well. Survival rate was normalized to the mean survival of the control wells in 5 mM glucose.

Primary cortical neurons were isolated and plated as described above. At 8 DIV, cortical neurons were washed once with phosphate-buffered saline (PBS), and feeding medium was replaced by NB-A supplemented with either 5 mM glucose or 5 mM lactate and 5 mM pyruvate for 16 hours. Neurons were then fixed in 4% paraformaldehyde (PFA) and permeabilized with 0.2% Triton X-100. The NeuN antibody (1:1000) was used to specifically target neurons. The total cell population was determined using Hoechst staining (1:2000). Neuronal population corresponds to the ratio between NeuN-positive cells and the number of Hoechst-positive cells.

#### Production and application of lentiviral particles

Lentiviral particles were produced by The Hope Center Viral Vectors Core at Washington University School of Medicine. Blue fluorescent protein (BFP) expression driven by cytomegalovirus promoter on pLKO.1-TRC mTagBFP2 was used to establish the precise volume of lentivirus needed to induce maximal transduction of cortical cultures. Lentivirus was added at 5 DIV, and media exchange was done after 2 days of infection. Gene expression analysis was carried out 7 to 10 days after viral transduction as described previously (*46*).

#### RNA isolation and quantitative PCR

RNeasy Mini Kits (QIAGEN, 74104) were used for total RNA extraction from primary dissociated cortical neuron cultures. Reverse transcription was performed to generate cDNA using an All-In-One 5X RT Master Mix (ABM-G592) following the manufacturer’s instructions. Using cDNA as the template and either TaqMan Fast Advanced Master Mix (Applied Biosytems, 4444557) or PowerUpTM SYBRTM Green Master Mix (Thermo Fisher Scientific, A25742), qPCR was set up. The ΔΔCt method was used to normalize relative mRNA expression to housekeeping genes, namely, *gapdh* or β*-actin*.

#### Western blotting

Neuronal extraction buffer (Thermo Fisher Scientific, 87792) mixed with 1X protease inhibitor cocktail and 0.1 mM phenylmethylsulfonyl fluoride (PMSF) was used to solubilize cultured cortical cells and brain tissues. For protein quantification, Bicinchoninic Acid (BCA) assay kit (BioVision), BioTek Synergy plate reader, and Gen5 software (BioTek Instruments, Winooski, VT) were used. Immunoblotting was conducted using a 12% SDS gradient polyacrylamide gel followed by transfer on polyvinylidene difluoride membrane. The primary antibodies used in this paper are as follows: ATPase5β (1:1000; Sigma-Aldrich, HPA001520), Sirt3 (1:1000; Cell Signaling Technology, 5490S), and anti–acetyl lysine (1:1000; Cell Signaling Technology, 9441S).

#### Immunoprecipitation of MPC1 from mouse brain

Cortical tissues from *Sirt3^+/+^* and *Sirt3^−/−^* mouse brains (five animals per genotype, 6 to 10 weeks old, mixed sex) were homogenized in immunoprecipitation (IP) buffer [15 mM NaCl, 25 mM tris base, 1 mM EDTA (0.19 g), 0.2% NP-40 (1 ml), 10% glycerol, 0.2 mM NaVO_3_, 0.5 mM NaF, 0.1 mM PMSF, and 1X protease inhibitor cocktail] and sonicated. Homogenates were then centrifuged for 30 min at 4°C, and the supernatant was precleared using Protein A agarose beads (30 μl) for 2 hours at 4°C. The beads were spun down, and the supernatant was incubated with normal rabbit IgG (3 μg; MilliporeSigma, 12-370) and MPC1 antibody (gift of B. Finck; 3 μg) overnight at 4°C. Protein A agarose beads (30 μl) were then added and incubated for 4 hours at 4°C. The supernatant was collected as unbound fraction after centrifugation, and the beads were boiled at 100°C for 10 min in elution buffer (IP and 1x Laemmli buffer).

#### Generation of Sirt3 KD HEK293 cells with CRISPR-Cas9 gene editing

HEK293 cells were trypsinized and washed with PBS. The ribonucleoprotein complex was prepared using single guide RNA against Sirt3 (IDT), Cas9 protein (Alt-R S.p. Cas9 Nuclease V3, IDT, 1081058), and nucleofection kit SF (Lonza, V4XC-2012) as per the manufacturer’s protocol. Program DG-130 was used on 4D-Nucleofactor system (Lonza) for transfection.

#### Immunoprecipitation of MPC1 from Sirt3 KD HEK293 cells

Sirt3 KD HEK293 cells generated with CRISPR-Cas9 (see above) or expressing shRNA against *sirt3* were transfected with either WT MPC1-FLAG or MPC1-AA-FLAG constructs using Lipofectamine 2000 (Invitrogen, 11668019). After 24 hours of transfection, cells were harvested and lysed in IP buffer [15 mM NaCl, 25 mM tris base, 1 mM EDTA (0.19 g), 0.2% NP-40 (1 ml), 10% glycerol, 0.2 mM NaVO_3_, 0.5 mM NaF, 0.1 mM PMSF, and 1X protease inhibitor cocktail]. The supernatant was collected after centrifugation at 13,000 rpm for 20 min and incubated with anti-FLAG–conjugated magnetic beads. The beads were then pulled down by magnetic rack and washed thrice with the IP buffer. The protein was then eluted from the beads by boiling them in elution buffer (IP and 1x Laemmli buffer) at 100°C for 10 min.

#### Immunofluorescence and confocal microscopy

PFA (4%) was used for fixation of primary Hippocampal neurons. Neurons were then permeabilized with 0.2% Triton X-100, blocked with 5% bovine serum albumin (BSA) for 1 hour at room temperature (RT), and incubated at RT for 2 hours or overnight at 4°C with the following primary antibodies against: MPC1 (1:500; rabbit, Sigma-Aldrich, HPA045119), MPC2 (1:500; rabbit, Thermo Fisher Scientific, 20049-1-AP), and vGLUT1 (1:500; guinea pig; Sigma-Aldrich, AB5905). Coverslips were then incubated with the following secondary antibodies: anti-rabbit Alexa Fluor 568 (1:500; Thermo Fisher Scientific, A21428) and anti-goat Alexa Fluor 488 (1:500; Thermo Fisher Scientific, A11073) for 1 hour at RT, mounted with anti-fade mounting media (Thermo Fisher Scientific, P36965), and kept at 4°C until imaged. Confocal images were collected on a Zeiss LSM 880 Confocal Microscope at the Washington University Center for Cellular Imaging, which was purchased with support from the Office of Research Infrastructure Programs (ORIP), as part of the National Institutes of Health (NIH) Office of the Director under grant OD021629.

#### Live imaging of neurons and HEK293 cells

A custom-built laser-illuminated epifluorescence microscope with a U Plan Fluorite 40X Oil Objective [numerical aperture (NA), 1.30] and an Andor iXon Ultra 897camera cooled to −80° to −95°C was used for live imaging experiments. Coverslips were mounted up in a laminar flow perfusion chamber and perfused with Tyrodes buffer consisting of 119 mM NaCl, 2.5 mM KCl, 2 mM CaCl_2_, 2 mM MgCl_2_, 50 mM Hepes (pH 7.4), 5 mM glucose or 1.25 mM lactate, and 1.25 mM pyruvate supplemented with 10 μM 6-cyano-7nitroquinoxalibe-2, 3-dione (CNQX) and 50 μM d,l-2-amino-5-phosphonovaleric acid (APV) (both from Sigma-Aldrich) to inhibit post-synaptic responses in neurons (these were not applied to HEK293 experiments). In experiments using field stimulation, APs were triggered using platinum-iridium electrodes with 1-ms pulses, producing field potentials of around 10 V/cm. All imaging was performed at 37°C using an Okolab stage top incubator for temperature control. As a standard, 30 frames were recorded before the stimulus train was triggered.

#### Near-TIRF microscopy of hippocampal synapses

All experiments were conducted at 37°C within a whole-microscope incubator chamber (TOKAI HIT). Fluorophores were excited with a 488 laser (Cell CMR-LAS-488, Olympus) and monitored using an inverted TIRF-equipped microscope (IX83, Olympus) under a 150×/1.45 NA objective (UapoN). The Z-drift compensation system (IX3-ZDC) was used to ensure a constant position of the focal plane during imaging. Near-TIRF was achieved by adjusting the incident angle to 63.7°, which is near the critical angle of 63.63°. Images were acquired every 50 ms using a cooled Electron Multiplying Charge-Coupled Device (EMCCD) camera (iXon Life 888, ANDOR). Field simulation was performed by using a pair of platinum electrodes and controlled by the software via Master-9 stimulus generator (A.M.P.I.).

#### Live imaging of mitochondrial pyruvate uptake

HEK293 cells were transfected with a fluorescence-based pyruvate sensor, mt-PyronicSF (*23*) only (control), or together with shRNA against *sirt3* (Sirt3 KD) or *mpc1* (MPC1 KD). Cells were first imaged in the same Tyrodes buffer as described above but without CNQX and APV. After recording 50 frames, cells were perfused with Tyrodes supplemented with 10 mM pyruvate. Once the fluorescence intensity reached a maximal plateau, cells were reperfused with 0 mM pyruvate. Areas of the coverslip typically containing 5 to 10 transfected cells were randomly selected for imaging.

#### Measurement of mitochondrial membrane potential with TMRM staining

HEK293 cells were transfected either with pAAV-GFP construct only (control) or together with shRNA against *sirt3* (Sirt3 KD) or *mpc1* (MPC1 KD) using Lipofectamine 2000 (Thermo Fisher Scientific) and incubated at 37°C in a 95% air/5% CO_2_ humidified incubator. Before imaging, cells were washed twice with minimum essential medium (MEM) (Thermo Fisher Scientific) and stained with 25 nM TMRM for 20 min at 37°C. Cells were washed thrice with MEM, incubated in 5 mM TMRM, and imaged on a custom-built laser-illuminated epifluorescence microscope as described above. GFP-transfected cells were selected for analysis, and the intensity of TMRM was measured using ImageJ, normalized to the mean of control, and plotted using GraphPad prism v9.0.

#### Analysis of live imaging data

Image analysis was primarily performed using the ImageJ plugin Time Series Analyzer where ∼20 to 50 regions of interest (ROIs) of ∼2 μm corresponding to responding nerve terminals were selected, and fluorescence intensity was measured over time. HEK293 cells were analyzed by drawing ROIs corresponding to the entire cell volume.

#### Quantification of presynaptic ATP with Syn-ATP

Dual luminescence and fluorescence imaging of the presynaptic ATP reporter, Syn-ATP, was performed using a custom-built setup and analyzed with a semi-automatic platform as previously reported (*46*, *70*). pH correction of luminescence/fluorescence (L/F) values was performed by collecting parallel measurements using the cytosolic pH sensor cyto-pHluorin expressed in neurons, as previously described (*30*).

#### Quantification of presynaptic ATP with synapto.iATPSnFR2

Neurons expressing plenti.CamKII.(synapto).iATPSnFR2.A95A.A119L.miRFP670nano3 (*68*) were imaged with sequential acquisition in GFP and far red (miRFP670) channels at a cycle rate of 0.5 Hz. For analysis, two independent time series of GFP and far red images were generated in ImageJ, and about 25 to 30 ROIs (~2 μm) corresponding to synaptic boutons were selected to quantify fluorescence intensity in each channel. The ATP level was reported as the average ratio of fluorescence intensity in the GFP channel to the far red channel.

#### Quantification of SV retrieval block

SV retrieval in hippocampus neurons expressing vGLUT1-pHluorin was quantified as previously described (*34*). Briefly, Images were captured at a frame rate of 2 Hz, and neurons were electrically stimulated with a train of 100 AP at 10 Hz. For experiments with MPC inhibitors, neurons were incubated with 50 to 100 μM UK5099 or Zaprinast for 7 min before imaging. Endocytic time constants (τ) were determined by fitting fluorescence change (Δ*F*) after the stimulation to a single exponential decay (*71*). The proportion of Δ*F* left after two times the average endocytic time constant of the control (2τ) was divided by maximal Δ*F* at the end of stimulation (i.e., Δ*F*_2τ_/Δ*F*_max_) and was used to compute the fractional retrieval block in the endocytosis of vGLUT1-pH.

#### Quantification of release probability

Release sites were defined using a hierarchical clustering algorithm using built-in functions in MATLAB as described (*39*). We have previously shown that the observed clusters do not arise from random distribution of release events, but rather represent a set of defined and repeatedly reused release sites within the AZs.

#### Event detection and localization using mixture-model fitting

The release event detection and localization at subpixel resolution were performed as previously described (*39*) using MATLAB and the uTrack software package, which was made available by G. Danuser’s laboratory (*72*, *73*). Localization precision was determined directly from least-squares Gaussian fits of individual events as previously described (*41*).

#### Definition of AZ dimensions and center

The AZ size was approximated on the basis of the convex hull encompassing all vesicle fusion events in a given bouton. This measurement is in close agreement with the ultrastructural measurements of AZ dimensions (*39*). AZ center was defined as the mean position of all fusion events in a given bouton.

#### Metabolic profiling of cultured cortical neurons

Cortical neurons were plated at a density of 1 × 10^6^ cells per well in precoated six-well plates. At 7 DIV, medium was replaced with fresh media containing glucose (5 mM) or a mixture of lactate and pyruvate (5 mM each). Nutrient uptake was determined by the method described before (*74*, *75*) with minor modifications. Media were collected after cells were incubated for 24 hours. U-^13^C labeled nutrients (glucose, lactate, glutamine, and glutamate; Cambridge Isotope Laboratories) were added into the media as the internal standards. Media extraction and metabolite detection were conducted accordingly. The quantification was performed by calculating the ratio between the labeled peak and the unlabeled peak of the same metabolite in each media sample. The nutrient consumption rate was finally normalized by the cell numbers and proliferation rate.

#### Jugular vein catheterization

Male C57BL/6 mice were purchased from Charles River at 6 weeks of age. To perform infusion studies, a catheter (Instech, C20PU-MJV1301) was placed in the right jugular vein and connected to a vascular access button (Instech, VABM1B/25) implanted subcutaneously in the back of the mice. All catheter implantation surgeries were performed at the Hope Center for Neurological Diseases, Washington University. Mice were allowed to recover from surgery for at least 1 week before tracer infusion.

#### Intravenous infusion of mice with U^13^C-Pyruvatefor metabolomics analysis

On the day of the procedure, U^13^C-Pyruvate (CIL, CLM-2440-PK) was freshly prepared in saline at a concentration of 400 mM. The mice were weighed to calculate the tracer infusion rate and fasted at 9 am (ZT2). At approximately 2:00 p.m., the vascular access button of individual mice was connected to the infusion line with a swivel (Instech, SMCLA), tether (Instech, KVABM1T/25), and infusion pump (CHEMYX, Fusion 100 T). The infusion line was prefilled with 400 mM U^13^C-Pyruvate. Prime infusion was initiated at 1 μl/min per g for 2 min, followed by continuous infusion at 0.1 μl/min per g for 2 hours. Following completion of the pyruvate infusion, mice were anesthetized, and blood was collected by cardiac puncture. Tissues were subsequently collected as quickly as possible (in 10 min or less) following euthanasia and snap-frozen in liquid nitrogen. Tissues were stored at −80°C until processing for LC-MS analysis.

#### Metabolite extraction from serum and tissues

To extract metabolites from serum, 5 μl of serum was mixed with 295 μl of ice-cold methanol:acetonitrile:water (2:2:1), vortexed, and incubated at −20°C for 1 hour. The liver and brain tissues were ground by pestle and mortar with liquid nitrogen. Ground tissue mixed with ice-cold methanol:acetonitrile:water (2:2:1) and subjected to two cycles of 7 m/s (30 s per cycle) using an Omni Bead Ruptor Elute Homogenizer. For every 1 mg of tissue wet weight, 30 μl of extraction solvent was added. Samples were then incubated at −20°C for 1 hour to precipitate protein. Serum and tissue extracts were centrifuged at 20,000*g* and 4°C for 10 min, and the supernatant was transferred into LC-MS vials.

#### Metabolite measurement with LC-MS

Ultrahigh-performance LC (UHPLC)/MS was performed with a Thermo Scientific Vanquish Horizon UHPLC system interfaced with a Thermo Scientific Q Exactive Plus Orbitrap mass spectrometer. Metabolites were separated on a HILICON iHILIC-(P)-Classic column (100 mm × 2.1 mm, 5 μm). The injection volume was 5 μl. The column compartment was maintained at 40°C. The mobile-phase solvents were composed of: A = 20 mM ammonium bicarbonate, 2.5 μM medronic acid, 0.1% ammonium hydroxide in 95:5 water:acetonitrile; and B = 5:95 water:acetonitrile. The following linear gradient was applied at a flow rate of 250 μl min^−1^: 0 to 1 min, 90% B; 12 min, 35% B; 12.5 to 14.5 min, 25% B; 15 min, 90% B followed by a re-equilibration phase of 10 column volumes. Data were acquired in negative ion mode with the following settings: spray voltage, 2.8 kV; sheath gas, 45 Arb; auxiliary gas, 10 Arb; sweep gas, 2 Arb; capillary temperature, 250°C; aux gas temperature, 350°C; mass range, 65 to 975 Da; resolution, 140,000; automatic gain control (AGC) target, 1 × 10^6^; maximum injection time, 200 ms. LC-MS data were processed and analyzed with the open-source Skyline software (*76*). Naturally occurring isotope distributions were corrected as previously described (*77*). ^13^C-labeled metabolite levels were normalized to the amount of circulating tracer (^13^C labeled pyruvate in serum) (*78*) and plotted with GraphPad Prism. To correct for multiple comparison, raw *P* values were adjusted using the false discovery rate (FDR) approach using the two-stage step-up method of Benjamini, Krieger, and Yekutielii. An FDR adjusted *P* value < 0.01 was considered statistically significant.

#### Statistical analysis

GraphPad Prism v10.0 or MATLAB were used for statistical analysis. When comparing two sets of data, *P* values were determined using the two-tailed, unpaired *t* test; the nonparametric Mann-Whitney *U* test; or the Kolmogorov-Smirnov test. For more than two datasets, two-way analysis of variance (ANOVA) test was used. *P* values of less than 0.05 were deemed statistically significant. On the basis of power analysis and estimations of data variability derived from earlier findings and pilot research, the appropriate sample size was chosen. Grubb’s test in GraphPad Prism was used to identify and eliminate outliers from further analysis.
