# Supplementary material for: Mitochondrial pyruvate transport regulates presynaptic metabolism and neurotransmission
Source: Sci Adv. 2024 Nov 15;10(46):eadp7423. doi: 10.1126/sciadv.adp7423 (PMC11567002; doi:10.1126/sciadv.adp7423)
Supplement: Supplementary file 1 — Figs. S1 to S5 Tables S1 to S3 [file sciadv.adp7423_sm.pdf]

Science Advances
AAAS

Supplementary Materials for

Mitochondrial pyruvate transport regulates presynaptic metabolism and neurotransmission

Anupama Tiwari *et al.*

Corresponding author: Ghazaleh Ashrafi, ghazaleh@wustl.edu

Sci. Adv. **10**, eadp7423 (2024)
DOI: 10.1126/sciadv.adp7423

This PDF file includes:

Figs. S1 to S5
Tables S1 to S3

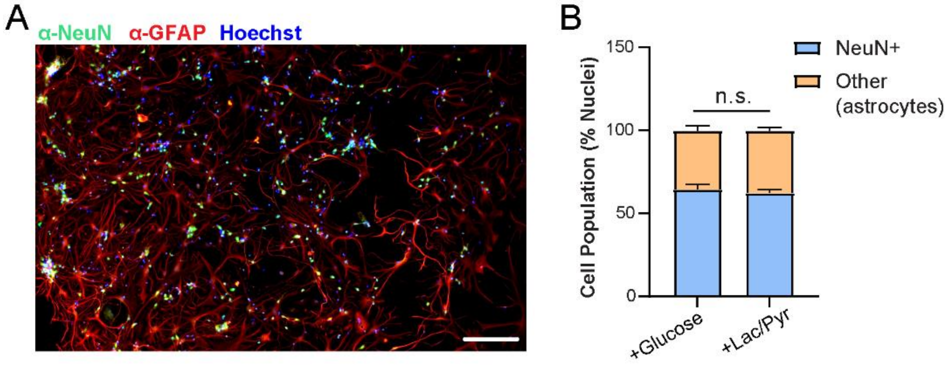

Fig. S1 (Related to Figure 1). Cell type composition and synaptic maturation in cortical neuronal cultures. (A) Immunostaining of cortical neuronal cultures with antibodies against NeuN (neuronal marker), GFAP (astrocyte marker), and Hoechst nuclear stain. Scale bar, 50 µm. (B) Quantification of percentage of total nuclei positively stained for NeuN in cultures treated with glucose or lactate/pyruvate for 12-16 hours. n = 20-21 fields of view (FOVs). Two-way ANOVA test. Error bars are SEM.

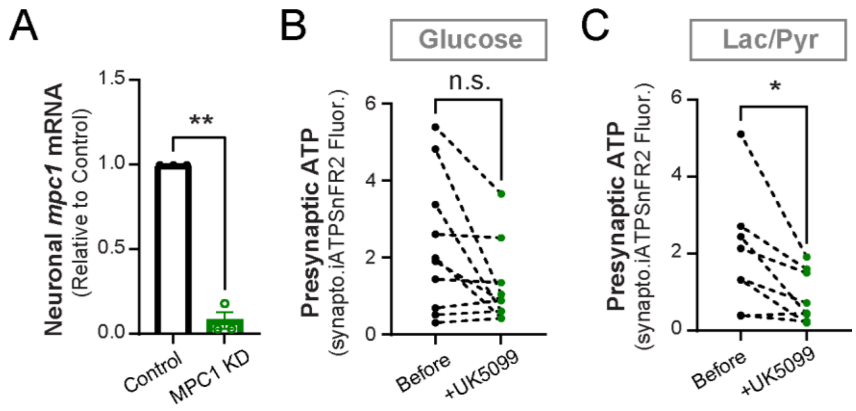

Fig. S2 (Related to Figure 2). Quantification of MPC1 knockdown efficiency in neurons and the effects of UK5099 on presynaptic ATP level. (A) Relative expression of mpc1 mRNA in control and MPC1 KD cortical neuronal cultures. Values are normalized to β-actin mRNA and expressed relative to the control. n= 3 cortical cultures. (B, C) Presynaptic ATP levels in hippocampal neurons expressing the ATP indicator synapto.iATPSnFR2. A95A.A119L.miRFP670nano3 before, and 8 minutes after treatment with UK5099 in glucose (5 mM) (B) or lactate/pyruvate (1.25mM each) (C). The ratio of GFP to miRFP fluorescence is averaged across terminals of a single neuron and plotted. n= 8-10 neurons. Mann-Whitney U test (A), Wilcoxon signed rank test (B, C). *p<0.05, **p<0.01. Error bars are SEM.

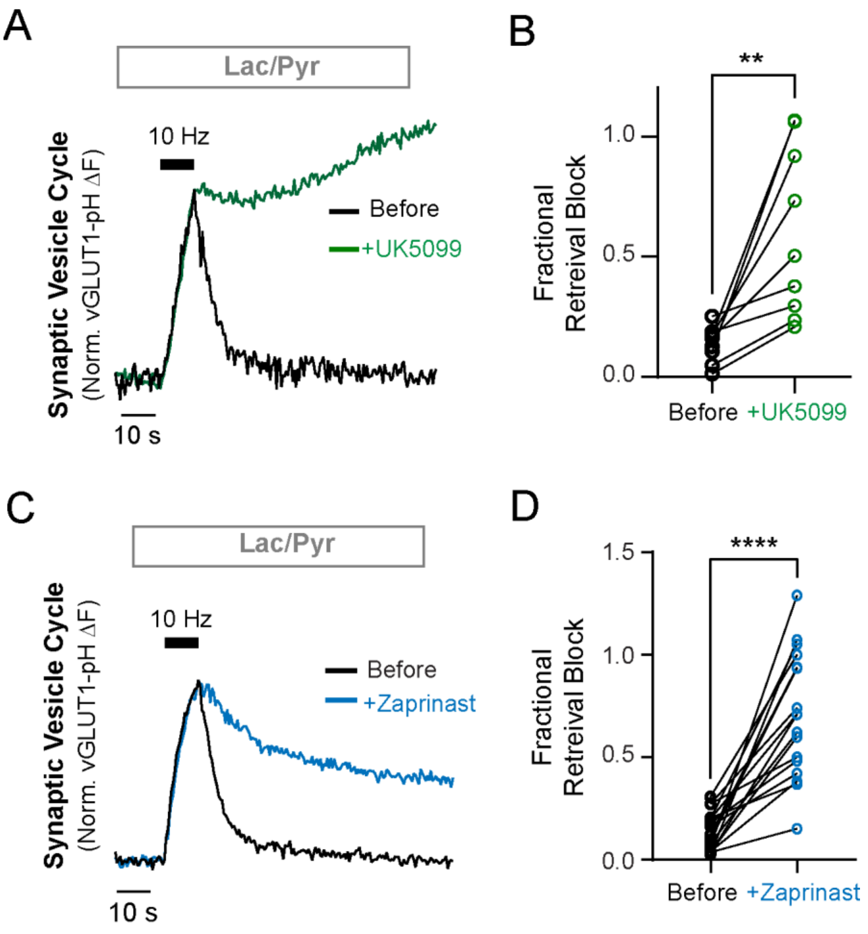

Fig. S3 (Related to Figure 3). Pharmacological inhibition of MPC impairs SV retrieval in hippocampal nerve terminals.

(A, C) Sample normalized vGLUT1-pH traces in hippocampal terminals electrically stimulated with 100 AP at 10 Hz before (control) and after treatment with MPC inhibitors UK5099 (A), or Zaprinast (C).

(B, D) SV retrieval quantified as fractional retrieval block from traces in A and C. n = 9 neurons (B). n = 10 neurons (D).

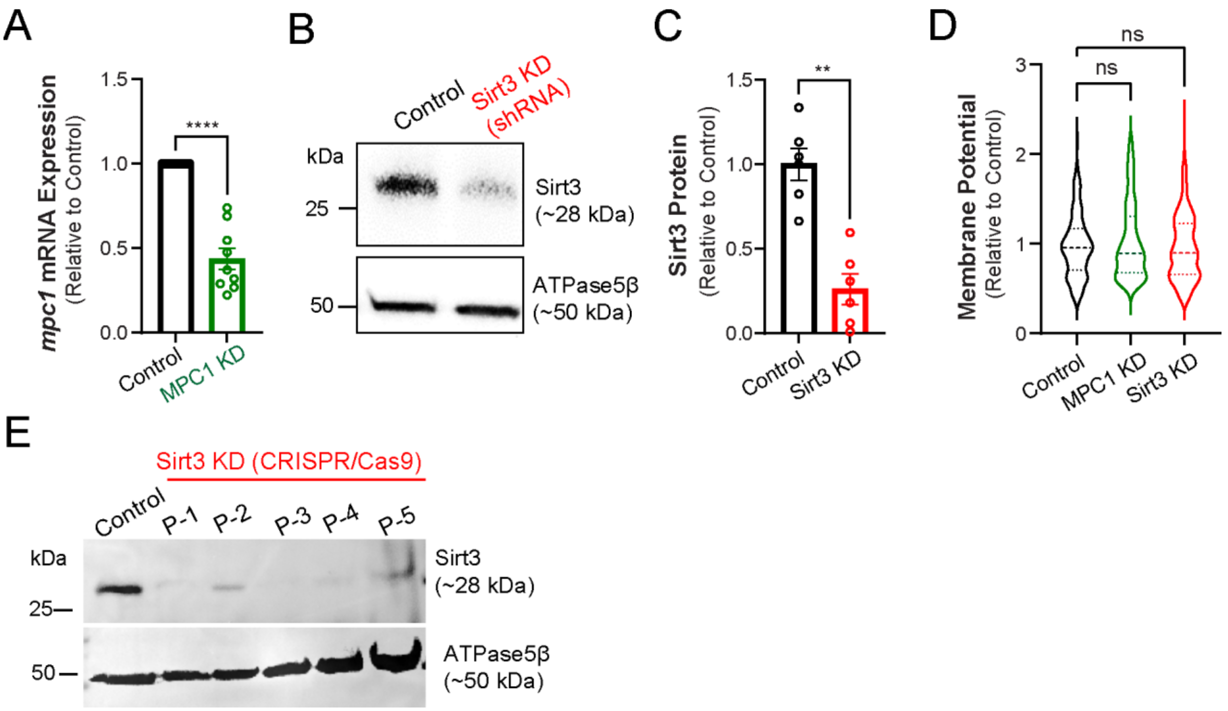

Fig. S4 (Related to Figure 4). Quantification of Sirt3 and MPC1 knockdown efficiency and characterization of mitochondrial membrane potential in HEK293 cells.

(A) Relative *mpc1* mRNA expression in control and MPC1 KD HEK293 cells. Values are normalized to *gapdh* mRNA and expressed relative to control. n =9 lysates. Average normalized mRNA level ± SEM: Control, 1.0 ± 0.0; MPC1 KD, 0.4 ± 0.06.

(B) Total protein lysate from control HEK293 cells and cells expressing shRNA against Sirt3 were immunoblotted for Sirt3 and mitochondrial ATPase5β.

(C) Relative expression of Sirt3 quantified from blots shown in panel E. Values are normalized to ATPase5β and expressed relative to the control. n = 6 (lysates/blots).

(D) Fluorescence intensity of TMRM staining in MPC1 KD and Sirt3 KD cells normalized to control (untransfected) cells. n = 318-388 (cells).

(E) Relative expression of Sirt3 protein in control HEK293 cells or Sirt3 KD cells created with CRISPR-Cas9 editing of *sirt3* gene, probed across successive culture passages (denoted as P). Mitochondrial ATPase5β was used as loading control. n=1 western blot, 6 total lysates. Mann-Whitney U test (A and C), Kruskal-Wallis test (D). ****$p < 0.0001$. Error bars are SEM.

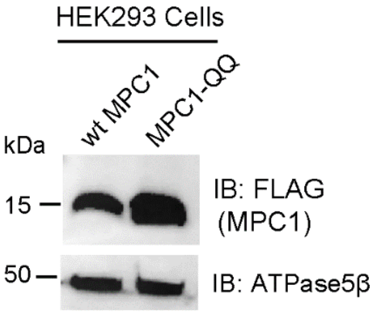

Fig. S5 (Related to Figure 5). Expression of MPC1 constructs in HEK293 cells. Total protein lysate from HEK293 cells expressing FLAG-tagged wildtype (wt) MPC1 or acetyl mimetic (MPC1-QQ) were immunoblotted for FLAG and the mitochondrial marker, ATPase5β.

Isotopologues	Pyruvate [m+0]	Pyruvate [m+1]	Pyruvate [m+2]	Pyruvate [m+3]	Pyruvate Total label	Lactate [m+0]	Lactate [m+1]	Lactate [m+2]	Lactate [m+3]	Lactate Total label
Serum_A	0.93	0.02	0.02	0.03	0.07	0.93	0.02	0.02	0.03	0.07
Serum_B	0.91	0.02	0.02	0.06	0.09	0.91	0.02	0.02	0.06	0.09
Serum_C	0.92	0.02	0.02	0.05	0.08	0.92	0.02	0.02	0.05	0.08
Serum_D	0.93	0.02	0.02	0.03	0.07	0.93	0.02	0.02	0.03	0.07
Brain_A	0.95	0.02	0.02	0.01	0.05	0.95	0.02	0.02	0.01	0.05
Brain_B	0.97	0.01	0.01	0.01	0.03	0.95	0.02	0.02	0.01	0.05
Brain_C	0.95	0.02	0.01	0.02	0.05	0.94	0.02	0.02	0.02	0.06
Brain_D	0.95	0.02	0.02	0.02	0.05	0.94	0.02	0.02	0.02	0.06
Liver_A	0.98	0.01	0.01	0.00	0.02	0.97	0.01	0.01	0.01	0.03
Liver_B	0.97	0.01	0.01	0.01	0.03	0.97	0.01	0.01	0.01	0.03
Liver_C	0.96	0.01	0.01	0.01	0.04	0.95	0.02	0.01	0.01	0.05
Liver_D	0.97	0.01	0.01	0.01	0.03	0.98	0.01	0.01	0.01	0.02

Isotopologues	Glucose [m+0]	Glucose [m+1]	Glucose [m+2]	Glucose [m+3]	Glucose [m+4]	Glucose [m+5]	Glucose [m+6]	Glucose Total label
Serum_A	0.89	0.05	0.04	0.03	0.00	0.00	0.00	0.11
Serum_B	0.90	0.04	0.03	0.03	0.00	0.00	0.00	0.10
Serum_C	0.87	0.06	0.04	0.03	0.00	0.00	0.00	0.13
Serum_D	0.89	0.04	0.04	0.03	0.00	0.00	0.00	0.11
Brain_A	0.95	0.00	0.05	0.00	0.00	0.00	0.00	0.05
Brain_B	0.96	0.00	0.01	0.00	0.00	0.00	0.02	0.04
Brain_C	0.99	0.00	0.01	0.00	0.00	0.00	0.00	0.01
Brain_D	0.99	0.00	0.01	0.00	0.00	0.00	0.00	0.01
Liver_A	0.97	0.02	0.01	0.01	0.00	0.00	0.00	0.03
Liver_B	0.97	0.02	0.01	0.01	0.00	0.00	0.00	0.03
Liver_C	0.89	0.05	0.03	0.02	0.00	0.00	0.00	0.11
Liver_D	0.97	0.01	0.01	0.01	0.00	0.00	0.00	0.03

Isotopologues	Hexose phosphate [m+0]	Hexose phosphate [m+1]	Hexose phosphate [m+2]	Hexose phosphate [m+3]	Hexose phosphate [m+4]	Hexose phosphate [m+5]	Hexose phosphate [m+6]	Hexose phosphate Total label
Serum_A	0.85	0.00	0.02	0.00	0.14	0.00	0.00	0.15
Serum_B	0.78	0.00	0.00	0.00	0.21	0.00	0.01	0.22
Serum_C	0.58	0.00	0.00	0.05	0.37	0.00	0.00	0.42
Serum_D	0.78	0.00	0.00	0.00	0.21	0.00	0.00	0.22
Brain_A	1.00	0.00	0.00	0.00	0.00	0.00	0.00	0.00
Brain_B	0.99	0.00	0.00	0.00	0.00	0.00	0.00	0.01
Brain_C	0.99	0.00	0.01	0.00	0.00	0.00	0.00	0.01
Brain_D	0.99	0.00	0.00	0.00	0.00	0.00	0.00	0.01
Liver_A	0.99	0.00	0.01	0.00	0.00	0.00	0.00	0.01
Liver_B	0.99	0.00	0.01	0.00	0.00	0.00	0.00	0.01
Liver_C	0.99	0.00	0.01	0.00	0.00	0.00	0.00	0.01
Liver_D	0.99	0.00	0.01	0.00	0.00	0.00	0.00	0.01

Isotopologues	Fructose Bis-phosphate [m+0]	Fructose Bis-phosphate [m+1]	Fructose Bis-phosphate [m+2]	Fructose Bis-phosphate [m+3]	Fructose Bis-phosphate [m+4]	Fructose Bis-phosphate [m+5]	Fructose Bis-phosphate [m+6]	Fructose Bis-phosphate Total label
Serum_A	0.72	0.00	0.00	0.00	0.08	0.12	0.08	0.28
Serum_B	0.48	0.05	0.07	0.00	0.21	0.13	0.06	0.52
Serum_C	0.06	0.00	0.14	0.00	0.04	0.00	0.77	0.94
Serum_D	0.37	0.00	0.13	0.00	0.22	0.12	0.16	0.63
Brain_A	0.98	0.00	0.02	0.00	0.00	0.00	0.00	0.02
Brain_B	0.98	0.00	0.02	0.00	0.00	0.00	0.00	0.02
Brain_C	0.98	0.00	0.02	0.00	0.00	0.00	0.00	0.02
Brain_D	0.98	0.00	0.02	0.00	0.00	0.00	0.00	0.02
Liver_A	0.94	0.00	0.02	0.01	0.02	0.00	0.00	0.06
Liver_B	0.96	0.00	0.02	0.01	0.01	0.00	0.00	0.04
Liver_C	0.93	0.01	0.03	0.02	0.01	0.00	0.00	0.07
Liver_D	0.93	0.00	0.02	0.02	0.03	0.00	0.00	0.07

Isotopologues	Citrate [m+0]	Citrate [m+1]	Citrate [m+2]	Citrate [m+3]	Citrate [m+4]	Citrate [m+5]	Citrate [m+6]	Citrate Total label
Serum_A	0.92	0.05	0.02	0.01	0.00	0.00	0.00	0.08
Serum_B	0.94	0.03	0.01	0.01	0.00	0.00	0.00	0.06
Serum_C	0.94	0.04	0.02	0.01	0.00	0.00	0.00	0.06
Serum_D	0.93	0.04	0.02	0.01	0.00	0.00	0.00	0.07
Brain_A	0.83	0.11	0.06	0.01	0.00	0.00	0.00	0.17
Brain_B	0.85	0.10	0.04	0.00	0.00	0.00	0.00	0.15
Brain_C	0.84	0.10	0.05	0.01	0.00	0.00	0.00	0.16
Brain_D	0.84	0.10	0.05	0.01	0.00	0.00	0.00	0.16
Liver_A	0.93	0.05	0.01	0.01	0.00	0.00	0.00	0.07
Liver_B	0.93	0.04	0.02	0.01	0.00	0.00	0.00	0.07
Liver_C	0.94	0.04	0.02	0.01	0.00	0.00	0.00	0.06
Liver_D	0.94	0.04	0.01	0.00	0.00	0.00	0.00	0.06

Isotopologues	Succinate [m+0]	Succinate [m+1]	Succinate [m+2]	Succinate [m+3]	Succinate [m+4]	Succinate Total label	Malate [m+0]	Malate [m+1]	Malate [m+2]	Malate [m+3]	Malate [m+4]	Malate Total label
Serum_A	0.88	0.04	0.08	0.00	0.00	0.12	0.93	0.04	0.02	0.01	0.00	0.07
Serum_B	0.60	0.12	0.19	0.00	0.09	0.40	0.93	0.04	0.02	0.01	0.00	0.07
Serum_C	0.84	0.02	0.07	0.00	0.06	0.16	0.93	0.04	0.02	0.01	0.00	0.07
Serum_D	0.89	0.00	0.09	0.00	0.01	0.11	0.94	0.04	0.02	0.01	0.00	0.06
Brain_A	0.84	0.12	0.03	0.00	0.00	0.16	0.86	0.10	0.04	0.00	0.00	0.14
Brain_B	0.86	0.11	0.03	0.00	0.00	0.14	0.88	0.08	0.03	0.00	0.00	0.12
Brain_C	0.85	0.11	0.04	0.00	0.00	0.15	0.87	0.09	0.03	0.00	0.00	0.13
Brain_D	0.85	0.11	0.04	0.00	0.00	0.15	0.87	0.09	0.04	0.00	0.00	0.13
Liver_A	0.93	0.05	0.02	0.00	0.00	0.07	0.93	0.05	0.02	0.00	0.00	0.07
Liver_B	0.92	0.05	0.02	0.00	0.00	0.08	0.93	0.04	0.02	0.01	0.00	0.07
Liver_C	0.94	0.04	0.02	0.00	0.00	0.06	0.94	0.04	0.02	0.01	0.00	0.06
Liver_D	0.93	0.05	0.01	0.00	0.00	0.07	0.94	0.04	0.02	0.00	0.00	0.06

Isotopologues	Glutamate [m+0]	Glutamate [m+1]	Glutamate [m+2]	Glutamate [m+3]	Glutamate [m+4]	Glutamate [m+5]	Glutamate Total label	Aspartate [m+0]	Aspartate [m+1]	Aspartate [m+2]	Aspartate [m+3]	Aspartate [m+4]	Aspartate Total label
Serum_A	0.93	0.04	0.03	0.00	0.00	0.00	0.07	0.96	0.03	0.01	0.00	0.00	0.04
Serum_B	0.94	0.03	0.02	0.00	0.00	0.00	0.06	0.95	0.03	0.01	0.01	0.00	0.05
Serum_C	0.95	0.03	0.02	0.00	0.00	0.00	0.05	0.97	0.02	0.01	0.00	0.00	0.03
Serum_D	0.94	0.04	0.02	0.00	0.00	0.00	0.06	0.95	0.03	0.01	0.00	0.00	0.05
Brain_A	0.84	0.10	0.06	0.00	0.00	0.00	0.16	0.88	0.08	0.04	0.00	0.00	0.12
Brain_B	0.86	0.09	0.05	0.00	0.00	0.00	0.14	0.89	0.08	0.03	0.00	0.00	0.11
Brain_C	0.85	0.10	0.05	0.00	0.00	0.00	0.15	0.88	0.08	0.03	0.00	0.00	0.12
Brain_D	0.84	0.10	0.05	0.00	0.00	0.00	0.16	0.88	0.08	0.03	0.00	0.00	0.12
Liver_A	0.95	0.03	0.02	0.00	0.00	0.00	0.05	0.95	0.04	0.01	0.00	0.00	0.05
Liver_B	0.95	0.02	0.02	0.00	0.00	0.00	0.05	0.95	0.03	0.02	0.00	0.00	0.05
Liver_C	0.96	0.02	0.02	0.00	0.00	0.00	0.04	0.95	0.03	0.01	0.00	0.00	0.05
Liver_D	0.96	0.02	0.02	0.00	0.00	0.00	0.04	0.96	0.03	0.01	0.00	0.00	0.04

Table S1 (Related to Figure 1). Isotopologues derived from $^{13}C_3$ pyruvate tracing in mouse tissues and serum. Data are presented as the fraction of total metabolites labeled with ^{13}C in different tissues from 4 mice (denoted as A, B, C, and D).

Pyruvate Consumption Rate (Fig 1F)

		Control media		Neuronal culture	
**Pyruvate Isotopologue**		**[m+0]**	**[m+3]**	**[m+0]**	**[m+3]**
	m/z	**87.0088**	**90.018865**	**87.0088**	**90.018865**
+Glucose	sample 1	21488957	16786251105	239244145	14888723009
	sample 2	25007884	15988839384	245954945	16859657350
	sample 3	19099959	15173338453	226370605	15378550766
+Lac/Pyr	sample 1	6878834864	15665260149	2321945972	15446645198
	sample 2	6169316360	14445753724	2213495973	15171144997
	sample 3	6226607930	14961289632	2444021211	16207574533

Metabolite Levels (Fig 1G)

		Glucose	Pyruvate	Lactate	Alanine	Citrate	a-ketoglutarate	Succinate	Fumarate	Malate	Aspartate	Glutamate	Glutamine	Proline
+Glucose	sample 1	2.80E+07	2.81E+08	1.74E+11	1.89E+09	1.01E+10	1.36E+08	1.57E+09	6.99E+08	1.12E+10	4.39E+09	1.51E+10	8.54E+09	2.83E+08
	sample 2	3.01E+07	2.80E+08	1.42E+11	1.91E+09	1.00E+10	1.23E+08	1.44E+09	4.26E+08	5.99E+09	3.93E+09	1.42E+10	7.74E+09	2.69E+08
	sample 3	1.79E+07	2.01E+08	1.04E+11	1.78E+09	8.87E+09	1.24E+08	1.22E+09	3.67E+08	5.19E+09	4.00E+09	1.39E+10	6.88E+09	2.67E+08
+Lac/Pyr	sample 1	3.62E+08	1.60E+09	1.02E+11	3.04E+09	1.30E+10	1.93E+09	1.28E+10	6.40E+08	9.52E+09	3.06E+09	1.99E+10	6.14E+09	3.80E+08
	sample 2	2.65E+07	1.84E+09	1.64E+11	2.54E+09	1.39E+10	6.82E+09	8.66E+09	7.60E+08	1.07E+10	3.25E+09	2.19E+10	6.84E+09	3.90E+08
	sample 3	9.02E+07	1.59E+09	1.08E+11	2.54E+09	1.25E+10	5.14E+08	9.69E+09	5.69E+08	8.39E+09	4.39E+09	1.72E+10	5.60E+09	3.19E+08

Table S2 (Related to Figure 1F, 1G). Metabolite pool sizes in primary cortical neurons supplied with glucose or lactate/pyruvate. Metabolite peak areas of pyruvate isotopologues m+0 and m+3 (Fig. 1F) and targeted metabolites (Fig. 1G) in neuronal cultures.

Figure 1	Sample number	Mean ± SEM	Statistical test
Figure 1C	4 male mice	see Supplementary Table 1	One-Way ANOVA
Figure 1E	3 (cultures); Glucose: 105 (wells), no Glucose: 108 (wells), Lac/Pyr: 107 (wells)	Glucose: 100 ± 1.5 ; no Glucose: 25.1 ± 1.4 ; Lac/Pyr: 57.6 ± 1.3	One-Way ANOVA
Figure 1F	1 (cultures); Glucose: 3 (wells); Lac/Pyr: 3 (wells)	Glucose: -0.00007746 ± 9.943e-006; Lac/Pyr: 0.001423 ± 0.0001220	Unpaired t-test
Figure 1G	1 (culture); Glucose: 3 (wells); Lac/Pyr: 3 (wells)	Glucose: Pyruvate (1.00 ± 0.15), Citrate (1.00 ± 0.04), Malate (1.00 ± 0.25), Succincate (1.00 ± 0.07); Lac/Pyr: Pyruvate (6.60 ± 0.31), Citrate (1.36 ± 0.04), Malate (1.28 ± 0.09), Succincate (7.34 ± 0.87)	Multiple unpaired t-tests
**Figure 2**	**Sample number**	**Mean ± SEM**	**Statistical test**
Figure 2F,G	3 (cultures), Ctrl: 21 (neurons), 19 (coverslips), MPC1 KD: 12 (neurons), 8 (coverslips)	Ctrl: 0.68 ± 0.10; MPC1 KD: 0.06 ± 0.08	Mann-Whitney U Test
Figure 2H,I	3 (cultures), 9 (neurons/coverslips)	After UK5099 (norm. to before): 0.6 ± 0.11	One sample t-test
**Figure 3**	**Sample number**	**Mean ± SEM**	**Statistical test**
Figure 3B	Ctrl: 3 (cultures)/ 16 (Coverslips) / 691 (Syn); +UK5099: 3 (cultures)/ 9 (Coverslips) / 230 (Syn)	Ctrl: 0.0763 ± 0.001; +UK5099: 0.0649 ± 0.001	Kolmogorov–Smirnov test
Figure 3C	Ctrl: 3 (Cultures) / 16 (Coverslips) / 732 (Syn); +UK5099: 3 (Cultures) / 9 (Coverslips) / 220 (Syn)	Ctrl: 9.57 ± 0.10; +UK5099: 6.30 ± 0.96	Kolmogorov–Smirnov test
Figure 3D	Ctrl: 3 (Cultures) / 16 (Coverslips) / 7,005 (Release site); +UK5099: 3 (Cultures) / 9 (Coverslips) / 1,705(Release Site)	Ctrl: 1.465 ± 0.009; +UK5099: 1.634 ± 0.0239	Kolmogorov–Smirnov test
Figure 3E	Ctrl: 3 (Cultures) / 16 (Coverslips) / 9,097 (events); +UK5099: 3 (Cultures) / 9 (Coverslips) / 2,786 (events)	Ctrl: 121.811 ± 0.338 ;+UK5099: 97.967 ± 1.415	Kolmogorov–Smirnov test
Figure 3F	Ctrl: 3 (cultures) / 16 (Coverslips) / 9,097 (events); +UK5099: 3 (cultures) / 9 (coverslips) / 2,786 (events)	Ctrl: 177.697 ± 0.623; +UK5099: 146.608 ± 0.873	Kolmogorov–Smirnov test
Figure 3I	Ctrl: 3 (cultures) / 16 (coverslips) / 691 (Syn); +UK5099: 3 (cultures) / 9 (coverslips) / 230 (syn)	Ctrl: 24.551 ± 0.977 (R^2 = 0.539 ± 0.003); +UK5099: 3.247 ± 0.61 (R^2 = 0.103 ± 0.008)	Kolmogorov–Smirnov test
Figure 3J	Ctrl: 3 (cultures) / 16 (coverslips) / 691 (Syn); +UK5099: 3 (cultures) / 9 (coverslips) / 230 (syn)	Ctrl: 0.828.241 ± 0.055 (R^2 = 0.454 ± 0.003); +UK5099: 0.487 ± 0.068 (R^2 = 0.068 ± 0.005)	Kolmogorov–Smirnov test
**Figure 4**	**Sample number**	**Mean ± SEM**	**Statistical test**
Figure 4A, B	5-27 (cultures), Ctrl: 50 (coverslips), 57 (FOV); MPC1 KD: 24 (coverslips)/ 33 (FOV); Sirt3 KD: 10 (coverslips)/ 18 (FOV)	Ctrl: 0.4 ± 0.02; MPC1 KD: 0.03 ± 0.02; Sirt3 KD: 0.21 ± 0.3	Kruskal-Wallis Test
Figure 4C, D	4 (IP/blots), Sirt3$^{+/+}$: 20 (mouse); Sirt3$^{-/-}$: 20 (mouse)	Ac-K intensity norm. to MPC1 intenstiy: Sirt3$^{+/+}$, 1.35 ± 0.44; Sirt3 $^{-/-}$, 2.10 ± 0.64. Ac-K intensity norm. to control: Sirt3$^{-/-}$, 1.6 ± 0.1	Paired t-test of Ac-K intensity norm. to MPC1 intenstiy
Figure 4F, G	6 (IP/blots)	Ac-K intensity norm. to MPC1-WT: MPC1 WT, 1.0 ± 0.0; MPC1 RR, 0.36 ± 0.08	One-Sample t-test
**Figure 5**	**Sample number**	**Mean ± SEM**	**Statistical test**
Figure 5B,C	10-27 (cultures), Ctrl: 50 (coverslips), 58 (FOV); MPC1 KD: 24 (coverslips)/ 33 (FOV); KD + wt MPC1: 19 (coverslips), 24 (FOV); KD + MPC1-QQ: 21 (coverslips), 22 (FOV). 12-15 cells/ FOV	Ctrl: 0.4 ± 0.02; MPC1 KD: 0.03 ± 0.02; KD + wt MPC1: 0.3 ± 0.03; KD + MPC1-QQ: 0.16 ± 0.05	Kruskal-Wallis Test
Figure 5E, F	3-13 (cultures), Ctrl: 46 (coverslips), 81 (neurons) ; MPC1 KD: 35 (coverslips), 71 (neurons); KD + wt MPC1: 24 (coverslips), 25 (neurons); KD + MPC1-QQ: 14 (coverslips), 16 (neurons)	Ctrl: 0.14 ± 0.01; MPC1 KD: 0.39 ± 0.03; KD + wt MPC1: 0.12 ± 0.02; KD + MPC1-QQ: 0.71 ± 0.15	Kruskal-Wallis Test
**Figure S1**	**Sample number**	**Mean ± SEM**	**Statistical test**
Figure S1A , B	2 (cultures), Glucose: 4 (coverslips), 21 (FOV); Lac/Pyr: 4 (coverslips), 20 (FOV)	% NeuN$^+$: Glucose: 64.9 ± 2.8; Lac/Pyr: 62.4 ± 2.1	Two-way ANOVA
**Figure S2**	**Sample number**	**Mean ± SEM**	**Statistical test**
Figure S2A	3 (cultures)	Ctrl: 1.0 ± 0.0; MPC1 KD: 0.08 ± 0.04	Mann-Whitney U Test
Figure S2B	3 (cultures), 10 (coverslips/neurons)	Before: 2.3 ± 0.5; UK5099: 1.24 ± 0.33	Paired Wilcoxon Test
Figure S2C	3 (cultures), 8 (coverslips/neurons)	Before: 2.39 ± 0.7; UK5099: 1.0 ± 0.4	Paired Wilcoxon Test
**Figure S3**	**Sample number**	**Mean ± SEM**	**Statistical test**
Figure S3A, B	3 (cultures), 9 (coverslips/neurons)	Before: 0.14 ± 0.02; UK5099: 0.59 ± 0.11	Paired Wilcoxon Test
Figure S3C, D	3 (cultures), 17 (coverslips/neurons)	Before: 0.13 ± 0.02; Zaprinast: 0.7 ± 0.07	Paired Wilcoxon Test
**Figure S4**	**Sample number**	**Mean ± SEM**	**Statistical test**
Figure S4A	3 (cultures), Ctrl: 9 (wells), MPC1 KD:9 (wells)	Ctrl: 1 ± 0.0; MPC1 KD: 0.43 ± 0.06	Mann-Whitney U Test
Figure S4B, C	3 (cultures), Ctrl: 6 (wells), Sirt3 KD: 6 (wells)	Ctrl: 1 ± 0.0 Sirt3 KD: 0.26 ± 0.09	Mann-Whitney U Test
Figure S4D	4 (cultures), Ctrl: 7 (coverslips), 26 (FOV), 318 (cells); MPC1 KD : 5 (coverslips), 18 (FOV), 272 (cells), Sirt3 KD: 7 (coverslips), 25 (FOV), 388 (cells). 12-15 cells/ FOV	Ctrl: 1. ± 0.02, MPC1 KD: 1.0 ± 0.03, Sirt3 KD: 0.95 ± 0.01	Kruskal-Wallis Test
**Figure S5**	**Sample number**	**Mean ± SEM**	**Statistical test**
Figure S5A	1 (blot); wt MPC: 4 (lysates); MPC1-QQ: 4 (lysates)	N/A	N/A

Table S3 (Related to Figures 1-5, and S1-5). Statistical data for figures. FOV: field of view. Syn: synapse
